# Prediction Under Interventions: Evaluation of Counterfactual Performance Using Longitudinal Observational Data

**DOI:** 10.1097/EDE.0000000000001713

**Published:** 2024-04-18

**Authors:** Ruth H. Keogh, Nan Van Geloven

**Affiliations:** From the a Department of Medical Statistics, London School of Hygiene & Tropical Medicine, London, United Kingdom; b Department of Biomedical Data Sciences, Leiden University Medical Center, Leiden, the Netherlands.

**Keywords:** Calibration, Causal prediction, Discrimination, Model performance, Model selection, Predictions under interventions, Risk evaluation, Validation

## Abstract

Predictions under interventions are estimates of what a person’s risk of an outcome would be if they were to follow a particular treatment strategy, given their individual characteristics. Such predictions can give important input to medical decision-making. However, evaluating the predictive performance of interventional predictions is challenging. Standard ways of evaluating predictive performance do not apply when using observational data, because prediction under interventions involves obtaining predictions of the outcome under conditions that are different from those that are observed for a subset of individuals in the validation dataset. This work describes methods for evaluating counterfactual performance of predictions under interventions for time-to-event outcomes. This means we aim to assess how well predictions would match the validation data if all individuals had followed the treatment strategy under which predictions are made. We focus on counterfactual performance evaluation using longitudinal observational data, and under treatment strategies that involve sustaining a particular treatment regime over time. We introduce an estimation approach using artificial censoring and inverse probability weighting that involves creating a validation dataset mimicking the treatment strategy under which predictions are made. We extend measures of calibration, discrimination (c-index and cumulative/dynamic AUCt) and overall prediction error (Brier score) to allow assessment of counterfactual performance. The methods are evaluated using a simulation study, including scenarios in which the methods should detect poor performance. Applying our methods in the context of liver transplantation shows that our procedure allows quantification of the performance of predictions supporting crucial decisions on organ allocation.

Estimates of absolute risk of outcomes under different treatment choices conditional on patient characteristics are important for informing individual decisions in healthcare. This includes enabling patients to weigh the risks and benefits of different treatment options and informing the allocation of treatments that are subject to resource constraints, such as donor organs. Standard prediction models do not provide the necessary information as they target the observed outcome distribution in the population in which the prediction model was developed, typically including a mix of individuals with some who followed the treatment strategy of interest and others who did not.^1^ The task of obtaining individualized estimates of risks under specified treatment strategies has been referred to as “causal prediction,” “counterfactual prediction,” and “prediction under hypothetical interventions.” In this article, we use the term “prediction under interventions.”

The fundamental challenge in developing and evaluating predictions under interventions is that after a patient receives treatment and we observe their outcome, it is impossible to know what the counterfactual outcome would have been had they received an alternative treatment. Predictions under interventions can be obtained from data collected in randomized controlled trials and some interventional prediction models have been developed and evaluated in this way.^2,3^ However, randomized trials are not designed for this purpose and may be limited by strict inclusion criteria, small sample size, and short-term follow-up. Longitudinal observational data from sources such as electronic health records, cohort studies, and patient registries, which provide rich data on large numbers of individuals, are often the main sources for developing models for prediction under interventions. To address confounding, several causal inference methods that were originally proposed for estimating marginal treatment effects have recently been adapted to develop interventional predictions conditional on baseline characteristics.^1,4–6^ Methods that combine treatment effect estimates from trials with estimates of untreated risk from observational data have also been proposed.^7,8^

The focus of this article is performance evaluation, which is essential to inform the adoption of interventional prediction models in practice. Evaluation of prediction models involves comparing estimated risks from the model with observed outcomes in a validation dataset. Treatment strategies targeted by an interventional prediction model will differ from those that are observed for a subset of the individuals in an observational validation dataset, meaning that there is no observed analogue of estimated risks for assessing predictive performance. This renders existing methods for evaluating predictive performance unusable in this setting. A recent review indicated that 0 out of 13 identified studies on prediction under interventions assessed performance and it has been described as the most pressing problem in this field.^5,8^ Our goal is to assess the counterfactual performance of an interventional prediction model. This means assessing how well the predictions would match the data if all individuals in the validation data had followed the treatment strategy under which predictions have been made.

Prior works described approaches for counterfactual evaluation of interventional prediction models in the setting of a point treatment and binary outcome.^9,10^ Boyer et al.^11^ recently formalized this and described extensions to longitudinal treatment strategies. These works did not cover time-to-event outcomes. An ad hoc approach has been used in the time-to-event setting where estimated risks obtained under a given treatment strategy are compared with the observed outcomes in the subset of individuals who actually followed that treatment strategy.^4^ As we show later, this “subset approach” is prone to selection bias.

In this article, we present the first general set of counterfactual performance measures for time-to-event outcomes. Our focus is on counterfactual performance evaluation using longitudinal observational data, and under treatment strategies that involve sustaining a particular treatment regime over time. The proposed approach is evaluated using a simulation study and we showcase its use by evaluating interventional prediction in the context of liver organ allocation.

## PREDICTION UNDER INTERVENTIONS

We begin by defining the target of estimation for prediction under interventions, that is, the estimand. Our focus is on the risk of an event up to a time horizon τ under specified longitudinal treatment strategies conditional on a set of predictors X. Earlier work targeted only untreated risk.^1,4,10,11^ However, interest may lie in prediction under other longitudinal treatment strategies. Deterministic static strategies are arguably most relevant for informing individual decision-making, and we let ${\underline{a}}_{0}=a_{0},a_{1},\ldots$ denote a static deterministic treatment strategy from time zero onwards. In the later simulation and illustration we consider the strategies of never initiating treatment (never treated, ${\underline{a}}_{0}=0$), and initiating treatment at time 0 and sustaining treatment thereafter (always treated, ${\underline{a}}_{0}=1$). We let $T^{{\underline{a}}_{0}}$ denote the counterfactual event time under strategy ${\underline{a}}_{0}$. The estimand is the risk of the event occurring before time τ under this strategy given the predictors X available at the time of making the prediction:


$$\begin{array}{l} R^{{\underline{a}}_{0}}(\tau |X)=Pr(T^{{\underline{a}}_{0}}\leq \tau |X). \end{array}$$ (1)

An overview of how $R^{{\underline{a}}_{0}}(\tau |X)$ can be estimated from longitudinal observational data using marginal structural model and cloning-censoring-weighting approaches is given in eAppendix 1; http://links.lww.com/EDE/C122. For the remainder of the article, we assume that there exists a model that can be used to estimate $R^{{\underline{a}}_{0}}(\tau |X)$ for a new individual.

## COUNTERFACTUAL PERFORMANCE ASSESSMENT

### Validation Data

We assume that an external validation dataset with n individuals is available for assessing the counterfactual predictive performance of an interventional prediction model. The validation data should be from a population reflecting that in which the model is targeted for use.^12^ It is assumed to be observational and with a longitudinal structure in which each individual is observed at regular time points (e.g., study visits) k=0,1,… up to the event or censoring time, which is observed in continuous-time. It must include the predictors X plus any variables additionally needed, such that these in combination with X form a valid adjustment set sufficient to control for confounding of the treatment-outcome association, including potential time-dependent confounding.

**FIGURE 1.**
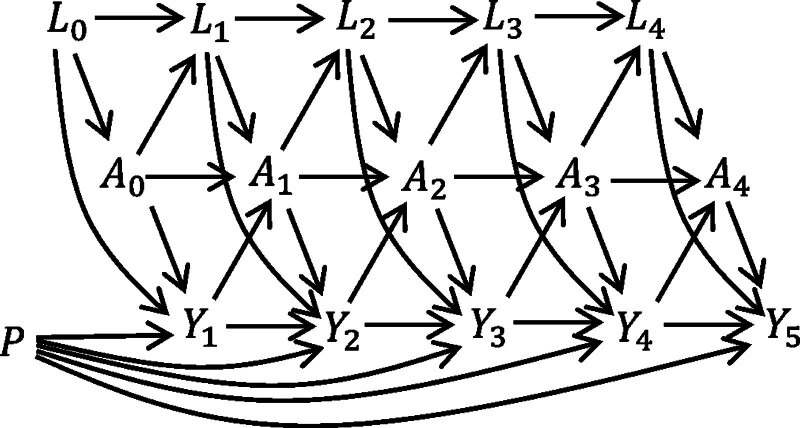
Directed acyclic graph (DAG) illustrating relationships between treatment A, time-dependent covariates L, baseline prognostic variables P, and discrete-time outcome Y. The DAG is illustrated for a discrete-time setting where $Y_{k}=I(k-1<T\leq k)$ is an indicator of whether the event occurs between visits k−1 and k. If the DAG is extended by adding a series of small time intervals between each visit, at which events are observed, then we approach the continuous-time setting. The covariates $L_{k}$ are time-dependent confounders as they inform treatment initiation or continuation, are predictive of the outcome, and are also affected by past treatment.

We let T and C denote event and censoring times, measured relative to the time from which a prediction would be made. For individual i the observed end of follow-up is $T_{i}^{*}=min(T_{i},C_{i})$, and $D_{i}$ is the event indicator. Time-dependent covariates $L_{k}$ and treatment status $A_{k}$ are recorded at each visit. We assume all individuals are untreated before time 0 ($A_{0^{-}}=0$), but may follow any treatment pattern thereafter. The structure of the validation dataset is illustrated in the directed acyclic graph (DAG) in Figure 1, which depicts the presence of time-dependent confounding by $L_{k}$. The predictors X may include all or a subset of the baseline confounders $L_{0}$, denoted $L_{0}^{*}$, in addition to variables P that predict the outcome but do not affect treatment status, that is, $X=\{L_{0}^{*},P\}$. The interventional prediction model is applied for each individual i=1,…,n in the validation data to obtain ${\hat{R}}^{{\underline{a}}_{0}}(\tau |X_{i})$, the estimate of risk up to time τ if they were to follow a treatment strategy ${\underline{a}}_{0}$.

### Artificial Censoring and Inverse Probability Weighting

Our proposed approach uses the observed validation data to generate modified validation datasets that emulate scenarios in which every subject followed each treatment strategy of interest. The first step involves applying artificial censoring in the validation data such that an individual’s follow-up is censored when they deviate from the strategy of interest, which could be at time 0. This is illustrated in eFigure 1; http://links.lww.com/EDE/C122 for the always treated (${\underline{a}}_{0}=1$) and never treated (${\underline{a}}_{0}=0$) strategies, but can be applied for any strategy for which predictions can be obtained provided there is a sufficiently large subgroup of individuals in the validation data who follow that strategy. The modified validation dataset restricted to follow-up under the strategy ${\underline{a}}_{0}$ is denoted $V^{{\underline{a}}_{0}}$. The second step involves weighting the individuals in $V^{{\underline{a}}_{0}}$ in such a way that it represents the population as if all individuals in the validation data had followed the treatment strategy ${\underline{a}}_{0}$. The weight at the time t is the inverse of the probability of not having been artificially censored up to that time, conditional on the covariate history required to control confounding, which in our DAG is ${\bar{L}}_{t}$.

In $V^{{\underline{a}}_{0}}$, let $C_{{\underline{a}}_{0}}$ denote the artificial censoring time and ${\tilde{T}}_{{\underline{a}}_{0}}=min(T^{*},C_{{\underline{a}}_{0}})$ the observed event time after artificial censoring with event indicator ${\tilde{D}}_{{\underline{a}}_{0}}=I(T^{*}<C_{{\underline{a}}_{0}})D$. The inverse probability of artificial censoring weights (IPACW) are:


$$\begin{array}{l} G_{{\underline{a}}_{0}}^{-1}(t|L)=\prod_{k=0}^{\left\lfloor t\right\rfloor }Pr{\left(C_{{\underline{a}}_{0}}>k|C_{{\underline{a}}_{0}}>k-1,{\bar{L}}_{k}\right)}^{-1}=\prod_{k=0}^{\left\lfloor t\right\rfloor }\frac{1}{Pr(A_{k}=a_{k}|{\bar{A}}_{k-1}={\bar{a}}_{k-1},{\bar{L}}_{k})}. \end{array}$$ (2)

The models used to derive the weights should be fitted in the validation data and not confused with any weight model fitted in the development data, as treatment assignments may be different in the development and validation datasets. Some estimators for evaluating predictive performance in the (noninterventional) prediction setting apply inverse probability of censoring weights to account for standard right censoring such as loss-to-follow-up or end-of-study (C above). We assume the standard censoring times do not depend on covariates (with $G_{c}(t)=P(C>t)$), but this could be extended to covariate-dependent censoring. Assuming independence between the artificial and standard censoring processes, we define the combined weight:


$$\begin{array}{l} G_{{\underline{a}}_{0}c}^{-1}(t|L)=G_{{\underline{a}}_{0}}^{-1}(t|L)\times G_{c}^{-1}(t). \end{array}$$ (3)

The validity of our approach, and of the estimates of counterfactual performance described below, relies on the causal assumptions of

1. Conditional sequential exchangeability: $T^{{\bar{A}}_{k-1},a_{-k}}⊥{\bar{L}}_{k},P,{\bar{A}}_{k-1},T\geq k$,
2. Consistency: $T=T^{{\underline{a}}_{0}}$ if ${\underline{A}}_{0}={\underline{a}}_{0}$_,_
3. Positivity: $0<Pr(A_{k}=a_{k}|{\bar{L}}_{k},P,{\bar{A}}_{k-1}={\bar{a}}_{k-1})<1$,

for all k. It also relies on the correct specification of the models used to estimate the weights.

## COUNTERFACTUAL PERFORMANCE MEASURES

We describe counterfactual measures of calibration, discrimination, and overall prediction error for validation of predictions under interventions. An overview of these measures in the standard prediction setting for time-to-event outcomes is given by McLernon et al.^13^ We specify estimands for each measure and describe estimators, which extend previously proposed estimators for the standard prediction setting, in particular by adding weights that depend on time-dependent covariates.

### Counterfactual Calibration

Calibration assessment focuses on how close estimates of risk by a particular time horizon τ from a prediction model are to the true underlying risks. For the assessment of counterfactual performance under the strategy ${\underline{a}}_{0}$, mean calibration compares the average estimated risk, ${\bar{R}}^{{\underline{a}}_{0}}(\tau )=\frac{1}{n}\sum_{i=1}^{n}{\hat{R}}^{{\underline{a}}_{0}}(\tau |X_{i})$, with the counterfactual outcome proportions by time τ under the strategy ${\underline{a}}_{0}$, $R_{Obs}^{{\underline{a}}_{0}}(\tau )$. An estimate of $R_{Obs}^{{\underline{a}}_{0}}(\tau )$, denoted ${\hat{R}}_{Obs}^{{\underline{a}}_{0}}(\tau )$, can be obtained by applying a weighted Kaplan–Meier analysis to $V^{{\underline{a}}_{0}}$, with weights $G_{{\underline{a}}_{0}}^{-1}(t|L)$. The estimates can be compared using the observed versus expected ratio ${\hat{R}}_{Obs}^{{\underline{a}}_{0}}(\tau )/{\bar{R}}^{{\underline{a}}_{0}}(\tau )$.

A stronger assessment of calibration is how well-estimated risks from the prediction model agree with the observed outcome proportions across the range of risk. To extend this to counterfactual performance assessment we divide the predictions ${\hat{R}}^{{\underline{a}}_{0}}(\tau |X_{i})$ (i=1,…,n) into G equal-sized groups (e.g., *G*=10), with the mean estimated risk in group g denoted ${\bar{R}}_{g}^{{\underline{a}}_{0}}(\tau )$. We then estimate the counterfactual outcome proportions in each group g=1,…,G, denoted ${\hat{R}}_{Obs,g}^{{\underline{a}}_{0}}(\tau )$, using a weighted Kaplan–Meier analysis for the group g in $V^{{\underline{a}}_{0}}$, with the same weights as above. The ${\hat{R}}_{Obs,g}^{{\underline{a}}_{0}}(\tau )$ (g=1,…,G) can then be plotted against ${\bar{R}}_{g}^{{\underline{a}}_{0}}(\tau )$ (g=1,…,G) to visually assess calibration.

### Counterfactual Discrimination

Concordance statistics for time-to-event outcomes, such as the c-index and the time-dependent area under the ROC curve (AUCt), compare pairs of individuals and evaluate whether the individual with the shorter survival time was assigned the higher risk by the model. In the presence of censoring, pairs in which one individual is censored before the other has an observed event are not “comparable,” and in a standard prediction context weighted estimators for the c-index and AUCt have been derived to address this.^14–17^ We extend these to allow the weights to incorporate time-dependent covariates.

We define the counterfactual c-index up to the time horizon τ under treatment strategy ${\underline{a}}_{0}$ as $C^{{\underline{a}}_{0}}(\tau )=Pr({\hat{R}}_{i}^{{\underline{a}}_{0}}(\tau |X_{i})>{\hat{R}}_{j}^{{\underline{a}}_{0}}(\tau |X_{j})|T_{i}^{{\underline{a}}_{0}}<T_{j}^{{\underline{a}}_{0}},T_{i}^{{\underline{a}}_{0}}\leq \tau )$, with ${\hat{R}}_{i}^{{\underline{a}}_{0}}(\tau |X_{i})$ and ${\hat{R}}_{j}^{{\underline{a}}_{0}}(\tau |X_{j})$ the predictions for a pair of individuals i and j. To estimate $C^{{\underline{a}}_{0}}(\tau )$, we use comparable pairs of individuals in $V^{{\underline{a}}_{0}}$. Time-dependent weights $G_{{\underline{a}}_{0}c}^{-1}(t|L)$ (Equation (3)) are applied to account for both artificial censoring and standard censoring. We propose the following weighted estimator:


$$\begin{array}{l} {\hat{C}}^{{\underline{a}}_{0}}(\tau )=\frac{\sum_{i=1}^{n}\sum_{j=1}^{n}I({\hat{R}}_{i}^{{\underline{a}}_{0}}(\tau |X_{i})>{\hat{R}}_{j}^{{\underline{a}}_{0}}(\tau |X_{j})){comp}_{{\underline{a}}_{0},ij}^{\left(1\right)}(\tau ){\hat{W}}_{{\underline{a}}_{0},ij}^{\left(1\right)}}{\sum_{i=1}^{n}\sum_{j=1}^{n}{comp}_{{\underline{a}}_{0},ij}^{\left(1\right)}(\tau ){\hat{W}}_{{\underline{a}}_{0},ij}^{\left(1\right)}} \end{array}$$ (4)

where ${comp}_{{\underline{a}}_{0},ij}^{\left(1\right)}(\tau )=I({\tilde{T}}_{{\underline{a}}_{0}i}<{\tilde{T}}_{{\underline{a}}_{0}j},{\tilde{T}}_{{\underline{a}}_{0}i}\leq \tau ,{\tilde{D}}_{{\underline{a}}_{0}i}=1)$ indicates whether the pair (i,j) is comparable up to time τ in $V^{{\underline{a}}_{0}}$, and ${\hat{W}}_{{\underline{a}}_{0},ij}^{\left(1\right)}={\hat{G}}_{{\underline{a}}_{0}c}^{-1}({\tilde{T}}_{{\underline{a}}_{0}i}^{-}|L_{i}){\hat{G}}_{{\underline{a}}_{0}c}^{-1}({\tilde{T}}_{{\underline{a}}_{0}i}|L_{j})$ is the weight of the pair, where ${\hat{G}}_{ac}^{-1}({\tilde{T}}_{{\underline{a}}_{0}i}^{-}|L_{i})$ is the left-hand limit of ${\hat{G}}_{ac}^{-1}({\tilde{T}}_{{\underline{a}}_{0}i}|L_{i})$.

Corresponding results for the cumulative/dynamic AUCt for prediction under interventions are in eAppendix 2; http://links.lww.com/EDE/C122.

Identification of these discrimination indices requires an extension of the positivity assumption to ensure a nonzero number of comparable pairs under the treatment strategies of interest.^11^

### Counterfactual Brier Score

The Brier score measures overall predictive performance and extends mean squared error to the time-to-event setting.^18^ The estimand for the Brier score for counterfactual performance under treatment strategy ${\underline{a}}_{0}$ is defined as $BS^{{\underline{a}}_{0}}(t)=E[(I(T^{{\underline{a}}_{0}}\leq t)-{\hat{R}}^{{\underline{a}}_{0}}(t|X{))}^{2}]$.

In $V^{{\underline{a}}_{0}}$, only observations not censored before t contribute to the calculation of $BS^{{\underline{a}}_{0}}(t)$. The proposed estimator weights these observations by the inverse of their probability of remaining uncensored:


$$\begin{array}{l} \hat{B}S^{{\underline{a}}_{0}}(t)=\frac{1}{n}\sum_{i=1}^{n}(I({\tilde{T}}_{{\underline{a}}_{0}i}\leq t)-{\hat{R}}_{i}^{{\underline{a}}_{0}}(t|X_{i}{))}^{2}W_{{\underline{a}}_{0}i}^{\left(2\right)}, \end{array}$$ (5)

with $W_{{\underline{a}}_{0}i}^{\left(2\right)}=\frac{I({\tilde{T}}_{{\underline{a}}_{0}i}\leq t,{\tilde{D}}_{{\underline{a}}_{0}i}=1)}{{\hat{G}}_{{\underline{a}}_{0}c}({\tilde{T}}_{{\underline{a}}_{0}i}|L_{i})}+\frac{I({\tilde{T}}_{{\underline{a}}_{0}i}>t)}{{\hat{G}}_{{\underline{a}}_{0}c}(t|L_{i})}$, representing weights for individuals whose event was observed before t and individuals observed to stay event-free by time t in $V^{{\underline{a}}_{0}}$. This extends a weighted estimator for baseline covariate-dependent censoring.^19^ The scaled Brier score under treatment strategy ${\underline{a}}_{0}$ is $BS_{scaled}^{{\underline{a}}_{0}}(t)=1-BS^{{\underline{a}}_{0}}(t)/BS_{0}^{{\underline{a}}_{0}}(t)$, where $BS_{0}^{{\underline{a}}_{0}}(t)$ is the Brier score of the null model. $BS_{0}^{{\underline{a}}_{0}}(t)$ can be estimated using (5), with ${\hat{R}}^{{\underline{a}}_{0}}(t|X_{i})$ replaced by the counterfactual outcome proportion up to time t, which can be estimated by a weighted sum of event indicators in $V^{{\underline{a}}_{0}}$ at time t, with ${\hat{G}}_{{\underline{a}}_{0}c}^{-1}(t)$ as weights.

## SIMULATION STUDY

### Simulation Plan

We evaluate the performance of the proposed measures of counterfactual predictive performance in a simulation study. In each simulation run, we first generate a development dataset and use this to derive a model for prediction under never treated and always treated strategies. Next, we generate a longitudinal observational validation dataset (eFigure 2; http://links.lww.com/EDE/C122), and obtain predictions under the never treated and always treated strategies using the development model. We estimate the predictive performance of the interventional predictions in the validation data, applying both the proposed approach to assess counterfactual performance and the ad hoc subset approach. These estimates are compared against predictive performance derived from two “perfect” validation datasets, one for each of the never treated and always treated strategies, generated in such a way that everyone followed the treatment strategy of interest.

Three main scenarios are considered. In scenario 1, the development and validation datasets are generated under the same model. In scenario 2, the development dataset has a higher baseline hazard than the validation dataset, but the form of the hazard model is otherwise the same. In scenario 3, the development and validation datasets are generated under the same model, but the predictions in the validation data are obtained using an error-prone version of $L_{0}$, denoted $L_{0}^{*}$. Scenarios 2 and 3 mimic settings where we expect poor counterfactual performance. In the three main simulation scenarios, the assumptions of consistency, positivity, conditional exchangeability, and correct specification of the weights model hold. In three additional scenarios, we examine where and how our method breaks down when one of these assumptions is violated. For all scenarios we consider data generating mechanisms using an additive hazards model and using a proportional hazards model. Full details of the simulation plan are given in eAppendix 3 and eTables 1–3; http://links.lww.com/EDE/C122 and descriptives of the resulting datasets in eAppendix 4 and eFigures 3 and 4; http://links.lww.com/EDE/C122.

R code for replicating the simulation is provided at https://github.com/survival-lumc/Validation_Under_Interventions.

### Simulation Results

**FIGURE 2.**
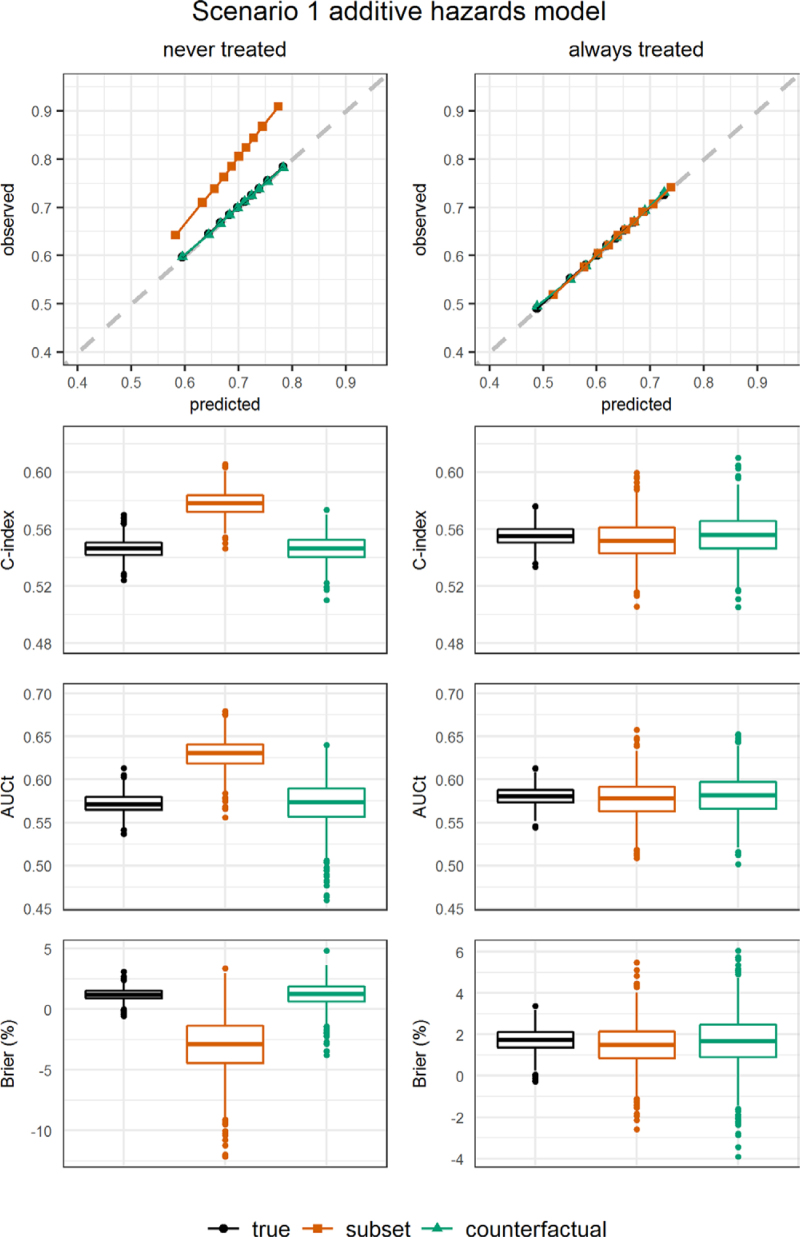
Simulation results: additive hazards model scenario 1. Left panel: for the never treated strategy. Right panel: for the always treated strategy. Performance measures were obtained from the perfect validation data (black dots) and estimated from the observational validation data using the subset approach (orange squares) and using the proposed artificial censoring + inverse probability weighted estimators of counterfactual performance (green triangles). Top row: calibration plot showing observed outcome proportions against mean estimated risks by time 5 within tenths of the estimated risks. Second row: c-index truncated at time 5. Third row: cumulative/dynamic area under the receiver operating characteristic curve at time 5. Bottom row: scaled Brier score at time 5.

**FIGURE 3.**
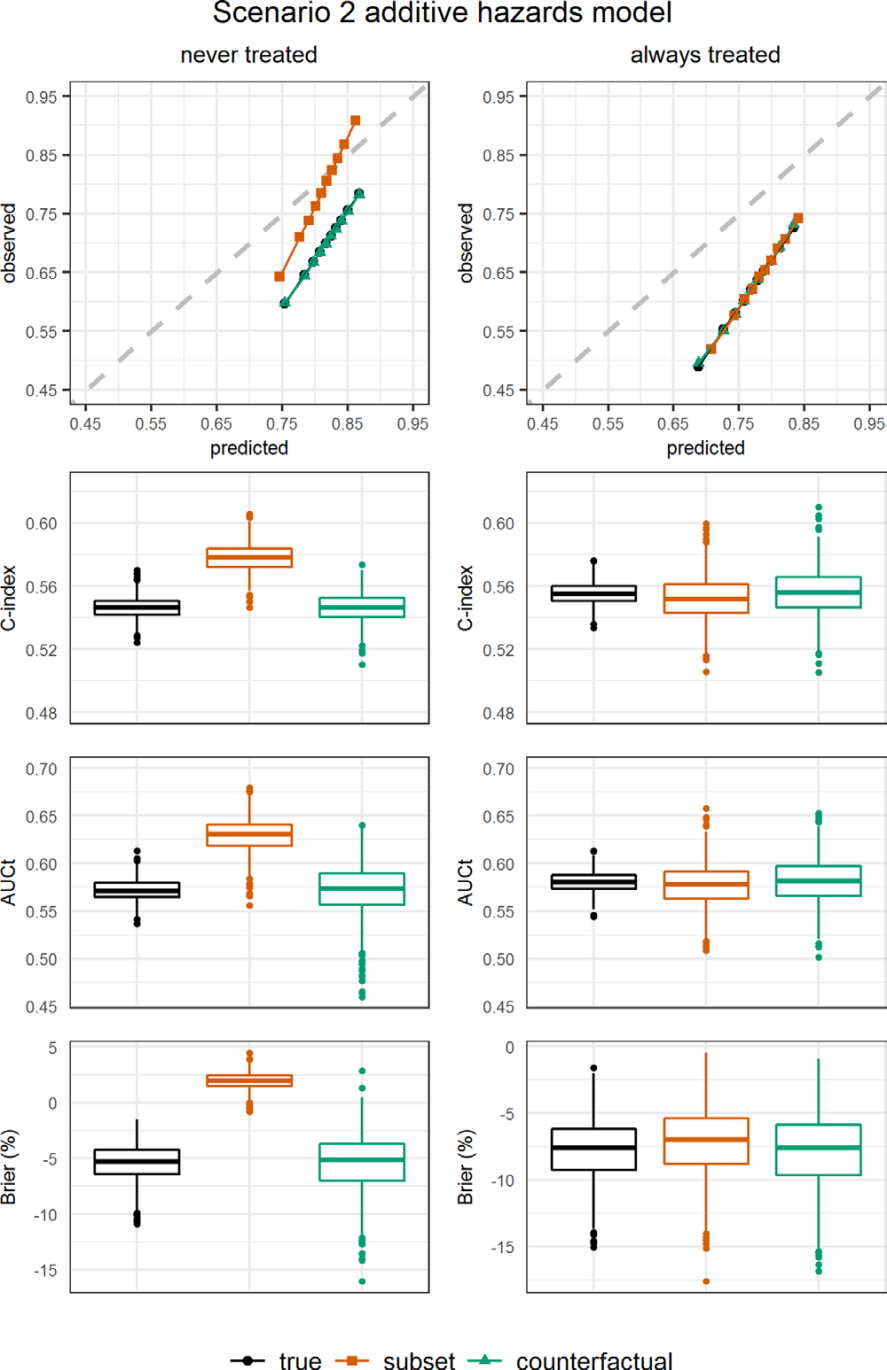
Simulation results: additive hazards model scenario 2. Left panel: for the never treated strategy. Right panel: for the always treated strategy. Performance measures were obtained from the perfect validation data (black dots) and estimated from the observational validation data using the subset approach (orange squares) and using the proposed artificial censoring + inverse probability weighted estimators of counterfactual performance (green triangles). Top row: calibration plot showing observed outcome proportions against mean estimated risks by time 5 within tenths of the estimated risks. Second row: c-index truncated at time 5. Third row: cumulative/dynamic area under the receiver operating characteristic curve at time 5. Bottom row: scaled Brier score at time 5.

**FIGURE 4.**
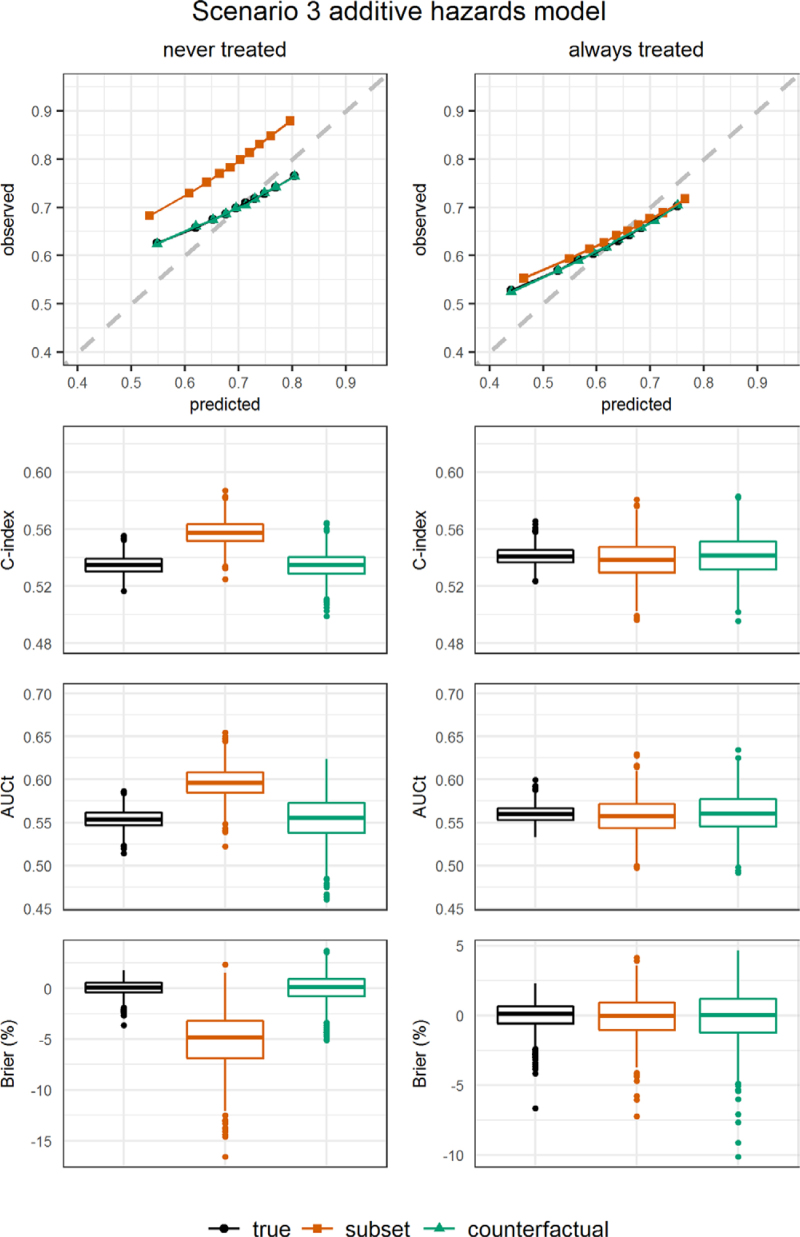
Simulation results: additive hazards model scenario 3. Left panel: for the never treated strategy. Right panel: for the always treated strategy. Performance measures were obtained from the perfect validation data (black dots) and estimated from the observational validation data using the subset approach (orange squares) and using the proposed artificial censoring + inverse probability weighted estimators of counterfactual performance (green triangles). Top row: calibration plot showing observed outcome proportions against mean estimated risks by time 5 within tenths of the estimated risks. Second row: c-index truncated at time 5. Third row: cumulative/dynamic area under the receiver operating characteristic curve at time 5. Bottom row: scaled Brier score at time 5.

Figures 2–4 show results for calibration, c-index, AUCt, and the scaled Brier score in scenarios 1–3, with data generated and analyzed using an additive hazards model. Corresponding numerical results are presented in eTables 4–6; http://links.lww.com/EDE/C122, where we also show the ratio of observed to estimated risks by time 5. Results from the other scenarios and from scenarios obtained from using a proportional hazards model to generate and analyze the data are presented in eAppendix 4 and eFigures 8–13; http://links.lww.com/EDE/C122 and eTables 7–11; http://links.lww.com/EDE/C122.

In scenario 1, where the developed prediction model is correctly specified and the development and validation data are generated from the same distributions, the calibration plot (top panel Figure 2) shows that our proposed method for counterfactual performance evaluation correctly assesses that estimated and observed outcome proportions lie on the diagonal (perfect calibration), with observed/expected ratios on average estimated very close to one (eTable 4; http://links.lww.com/EDE/C122). The subset approach wrongly suggested miscalibration for the predictions under the never treated strategy. The counterfactual evaluation of discrimination resulted in correct estimates of c-index and AUCt, whereas the subset approach overestimated these indices for the never treated strategy (middle panels Figure 2, eTable 4; http://links.lww.com/EDE/C122). The subset approach estimated a negative scaled Brier score on average in the never treated strategy (lower panel Figure 2) suggesting that the developed model was no better than a null model assigning the average risk to all subjects in the validation data, contrary to the true positive scaled Brier score. The proposed methods for counterfactual performance evaluation also showed unbiased results for the always treated strategy. Discussion of the size and direction of the biases when using the subset approach is presented in eAppendix 4; http://links.lww.com/EDE/C122.

In scenario 2, the generated risks were based on a higher baseline hazard in the development data compared with the validation data. The counterfactual measures of calibration correctly detected the resulting overestimation of estimated risks compared to observed outcome proportions, with the calibration curves lying below the diagonal (Figure 3), the observed/expected ratios being below one and negative scaled Brier scores for both treatment strategies (eTable 5; http://links.lww.com/EDE/C122). The subset approach gave estimated observed/expected ratios that are too high and for the never treated strategy it incorrectly suggested a positive scaled Brier score. Discrimination results are unaffected by changes in baseline hazard only, so the results for c-index and AUCt are equal to those in scenario 1.

In scenario 3 we used an error-prone version of $L_{0}$ when obtaining risk estimates in the validation data. The resulting lower levels of c-index, AUCt and scaled Brier score were correctly picked up by the counterfactual performance measures (Figure 4, eTable 6; http://links.lww.com/EDE/C122). The counterfactual calibration plot correctly showed that using the error-prone $L_{0}$ results in estimated risks that are too extreme, such that the very high and very low estimated risks correspond to observed outcome proportions that are closer to the average (top panel Figure 4). Averaged over all patients the risks are still well calibrated, with the observed/expected ratios around one. The subset approach again overestimates observed/expected ratios and discrimination indices in the never treated strategy.

Results for the other performance measures in scenarios 1–3 are presented in eFigures 5–7; http://links.lww.com/EDE/C122 and confirm the above observations.

As expected, the scenarios with deliberately introduced violations of causal assumptions in the validation data showed bias in the estimates of counterfactual performance. Bias varied across the different degrees of violations. Numerically, bias was modest in the counterfactual discrimination measures and was more pronounced in the estimates of the observed/expected ratio for calibration in some scenarios. Despite the violations, the bias of the counterfactual performance metrics was smaller than that of the naive subset approach in 58/64 (90%) of times in the additive hazards model scenarios (eTable 10; http://links.lww.com/EDE/C122).

## APPLICATION TO LIVER TRANSPLANTATION

We illustrate our methods with an application to liver transplantation, using data from the Scientific Registry of Transplant Recipients (SRTR). The SRTR data system includes data on all donors, wait-listed candidates, and transplant recipients in the United States, submitted by the members of the Organ Procurement and Transplantation Network. The Health Resources and Services Administration, US Department of Health and Human Services provides oversight to the activities of the Organ Procurement and Transplantation Network and SRTR contractors. The LSHTM Research Ethics Committee reviewed and approved the research plan.

We consider a composite outcome of death or removal from the transplant waitlist due to worsening health status. Our aim is to estimate for all wait-listed individuals at any given time their risk of the composite outcome up to 3 years under two intervention strategies: (1) receiving a liver transplant at that time; (2) not receiving a transplant at that time or in the future. The predictions under each intervention are to be conditional on the most recent measurements of individual characteristics. At the moment a new donor organ becomes available, such predictions would enable decision-makers to weigh the estimated risks of all wait-listed candidates under both strategies, which could inform organ allocation.

We used data on 43,190 individuals who joined the liver transplant waitlist between 1 January 2014 and 30 April 2019. Information recorded includes date of receiving a transplant, date of death, and date of and reason for removal from the waitlist, alongside longitudinal measurements of biomarkers, complications, and comorbidities. We created an analysis dataset that combines: (1) a dataset of individuals followed-up from transplant onwards; (2) datasets starting at a series of landmark times (from the time of joining the waitlist) restricted to individuals who remain on the waitlist and are untransplanted at the landmark time. The combined dataset was divided randomly into a 70% sample used for model development and a 30% sample used for the validation. Prediction models under the two interventions were fitted using the development dataset, applying an extension of methods used previously to estimate the effects of transplant to the prediction under the interventions setting.^20,21^ The validation methods proposed above were then used. Details about data set-up, model development, and how the validation methods were applied are given in eAppendix 5 and eTables 12–14; http://links.lww.com/EDE/C122.

**FIGURE 5.**
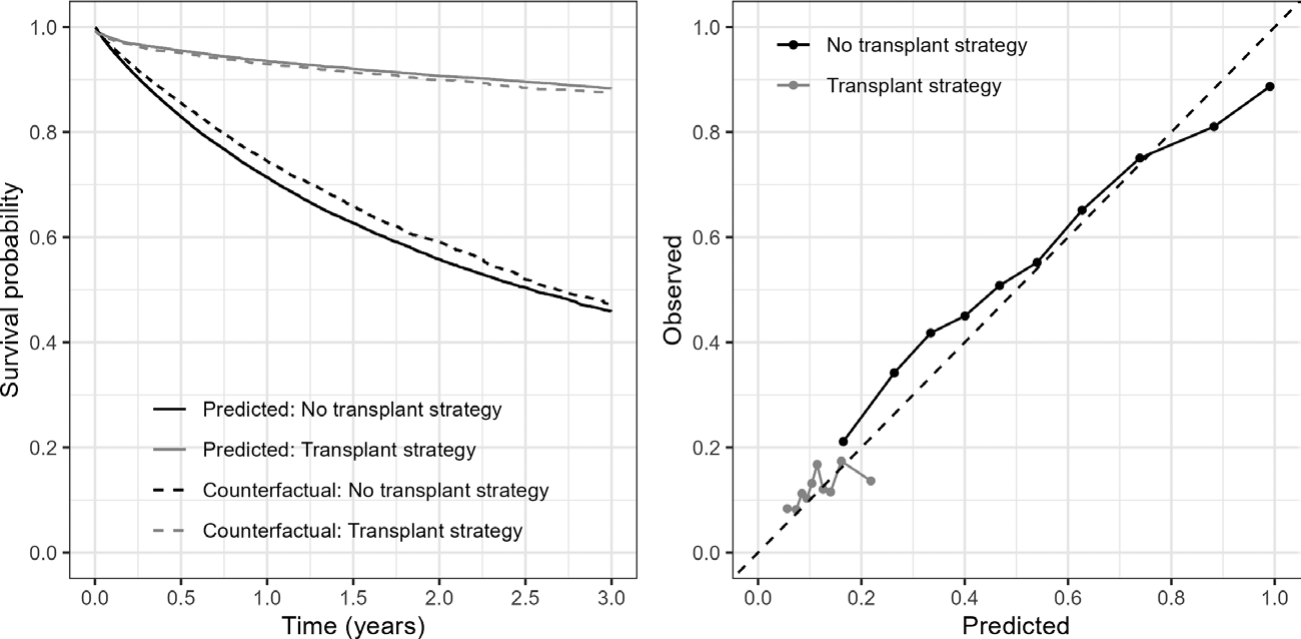
Liver transplant application: calibration of estimated risks under the no transplant and transplant strategies. Left: Plot showing the mean estimated survival curves up to 3 years under the two transplant strategies (solid lines), and the corresponding counterfactual survival curves (dashed lines), obtained using the artificial censoring plus inverse probability weighting approach. Right: Plot of counterfactual outcome proportions by 3 years against mean estimated risk by 3 years within 10 equal-sized groups of estimated risk under the two transplant strategies, showing the line of equality (dashed line).

Figure 5 shows calibration plots. Mean predicted survival curves differ substantially between the two intervention strategies, with mean estimated risk by 3 years being 54.1% under the no transplant strategy and 11.7% under the transplant strategy. The “observed” overall survival curves are close to the mean predicted curve, though the predicted curve under the no transplant strategy is slightly too low. Mean calibration is better for the transplant strategy. The comparison of mean risk estimates for τ=3 years within ten groups with the “observed” outcome proportions within each group suggests some overfitting for the no transplant strategy: low risks tending to be underestimated and high risks overestimated. A similar pattern is seen for the transplant strategy, for which we see a narrow range of predicted risks. The Table summarizes the other validation metrics. The c-index up to 3 years is 0.749 for prediction under the no transplant strategy and 0.561 for prediction under the transplant strategy. The AUCt’s are 0.781 and 0.552, and scaled Brier scores are 66.8% and 12.0%.

**TABLE.** Liver Transplant Application: Evaluation of Counterfactual Performance

	Strategy	
	No Transplant	Transplant
Calibration: observed/expected ratio based on risk by 3 years	0.983	1.060
Discrimination: C-index up to 3 years	0.749	0.561
Discrimination: AUCt at 3 years	0.781	0.552
Prediction error: scaled Brier score (%) at 3 years	66.8	12.0

AUCt, cumulative/dynamic area under the receiver operating characteristic curve.

The counterfactual validation approach allowed us to assess all relevant aspects of model performance. The results could inform improvements to these models or comparisons with alternative ones. The subset approach would have given an incorrect impression of the performance of the interventional prediction models (eTable 15; http://links.lww.com/EDE/C122 and eFigure 14; http://links.lww.com/EDE/C122).

## DISCUSSION

In this article, we proposed a new approach for counterfactual validation of predictions under interventions for time-to-event outcomes. We allow the use of longitudinal observational data with time-dependent confounding, and for interventions that involve sustaining a treatment over time. Our proposed approach is based on creating modified validation datasets that emulate scenarios in which every individual follows each treatment strategy of interest, through the use of artificial censoring and inverse probability weighting. We have provided the first general set of measures of counterfactual predictive performance for time-to-event outcomes, including measures of calibration, discrimination, and overall prediction error. Our simulation study showed that the proposed measures correctly capture true predictive performance, including detecting poor performance when they should. An application in the context of liver transplantation showed that our procedure allows quantification of the performance of predictions supporting crucial decisions on organ allocation.

Our validation approach relies on the correct specification of the models used to generate the inverse probability weights, and on the assumptions of consistency, positivity and conditional exchangeability. Simulation scenarios with violation of these assumptions showed the bias introduced when they do not hold. To avoid positivity violations and in general large uncertainty in estimates, it is advised to only consider treatment strategies that are followed by a sufficiently large subgroup in the validation data. Pragmatic descriptions of treatment strategies may help in this regard. In general, the censoring and weighting approach without a structural model is less efficient compared with approaches where treatment effects are modeled.^22^ Alternative approaches that employ a type of outcome modeling in the validation step have been proposed for counterfactual evaluation of binary outcome predictions.^10,11^ Extensions of our approach that allow relaxation of modeling assumptions, such as doubly robust approaches, are a priority for future work. In the longitudinal time-to-event setting, such approaches would require modeling of longitudinal data over time, in line with the g-formula approach to predictions under interventions used by Dickerman et al.^6^ We studied a setting in which the validation data have longitudinal information on treatment use and on time-fixed and time-dependent confounders, alongside the event or censoring time, but our approach can also be used in simpler settings, for example with point treatments or only time-fixed confounders. Our validation approach is also straightforward to extend to more complex treatment strategies, such as dynamic regimes. It is important to emphasize that validation of interventional predictions requires that the validation data includes not only the predictors, but also any additional variables required to control confounding, and information on starting and stopping of treatment.

In the simulation study, we imagined an external validation dataset, but many prediction studies rely initially on internal validation. In the application, we used a split sample approach. Further work is needed to demonstrate how our validation methods can be extended for use with cross-validation and bootstrapping. For example, in cross-validation, if each fold has a limited sample size then stable estimation of the weights could be challenging. In addition, methods for establishing sample size requirements are an area for future work, as are methods for model updating such as using recalibration. While methods for the development of interventional prediction models have been increasing, there has been very little focus on the validation of the resulting predictions. Assessment of counterfactual predictive performance is a pivotal step toward the implementation of interventional prediction models and may provide a novel instrument for model selection and tuning.

## ACKNOWLEDGMENTS

The data reported here have been supplied by the Hennepin Healthcare Research Institute (HHRI) as the contractor for the Scientific Registry of Transplant Recipients (SRTR). The interpretation and reporting of these data are the responsibility of the author(s) and in no way should be seen as an official policy of or interpretation by the SRTR or the US Government. The authors thank Ilaria Prosepe (Leiden University Medical Center, NL) for advice on the application.

This article was updated on July 19, 2024, with a correction for Formula 2 on page 331.

## Supplementary Material
