# Supplementary material for: Prediction Under Interventions: Evaluation of Counterfactual Performance Using Longitudinal Observational Data
Source: Epidemiology. 2024 Apr 18;35(3):329–39. doi: 10.1097/EDE.0000000000001713 (PMC11332371; doi:10.1097/EDE.0000000000001713)
Supplement: Supplementary file 1 [file ede-35-329-s001.pdf]

Prediction under interventions: evaluation of counterfactual performance using longitudinal observational data

Supplemental Digital Content

Keogh, Ruth H.[a] & Van Geloven, Nan[b †]

[a]Department of Medical Statistics, London School of Hygiene & Tropical Medicine, London, UK

[b]Department of Biomedical Data Sciences, Leiden University Medical Center, Leiden, NL

[†] The two authors contributed equally.

eAppendix 1 Development of interventional prediction models using observational data

Development data

A cohort of individuals is assumed to be available for development of a model for predictions under interventions. We let T denote the time to the event of interest, measured relative to the time point from which a prediction would be made, and C denotes the censoring time. For individual i the observed end of follow-up is $T_i^* = \min(T_i, C_i)$, and D_i is the event indicator. We focus on a setting in which each individual in the cohort is observed at regular time points (e.g. study visits) $k = 0, 1, \ldots$ up to the event or censoring time. Time-dependent covariates L_k and treatment status A_k are recorded at each visit. Additional prognostic variables P are recorded at baseline. Events and censorings are assumed to be observed in continuous time (i.e. not just at the visit times). We let $X = \{L_0^*, P\}$ denote the baseline characteristics to be used when estimating the risk, where L_0^* denotes a subset of L_0, which could be all or none of L_0. We focus on the setting where all individuals are untreated before time 0 ($A_{0^-} = 0$) (incident users), but they may follow any treatment pattern from time zero onwards, with the treatment pattern followed depending on both baseline and time-dependent covariates L.

The assumed data structure is as illustrated in the DAG in Figure 1 in the main text. That DAG refers to the assumed structure of the validation data, but we assume it also applies to the development data here. However, we emphasise that there is no requirement for the development and validation datasets to be of the same form. Main Figure 1 depicts the presence of time-dependent confounding, which arises when there are time-dependent covariates predictive of the outcome that also inform treatment initiation or continuation, and which are affected by past treatment.

Development methods

Below we outline the MSM-IPTW approach and cloning-censoring-weighting approach. Our focus is on two sustained treatment strategies: (i) never initiating treatment, which we refer to as the *never treated* strategy; (ii) initiating treatment at time 0 and sustaining treatment thereafter, which we refer to as the *always treated* strategy.

Development using MSM-IPTW

The MSM-IPTW approach involves first specifying a marginal structural hazard model, which is a model for the hazard for counterfactual event times given a longitudinal treatment strategy. Because we wish to obtain estimates of risk conditional on baseline covariates X, these should

also be conditioned on in the MSM. We let $T^{\underline{a}_0}$ denote the counterfactual event time under the treatment strategy $\underline{a}_0$, which denotes treatment status from time 0 onwards. The MSM for the hazard can take any form. Cox models[1] are often used, and under this model a general form for the MSM for the hazard under the treatment strategy $\underline{a}_0$ is

$$h_{T^{\underline{a}_0}}(t|X;\boldsymbol{\beta}) = h_0(t)\exp\left\{g(\bar{a}_{\lfloor t\rfloor};\beta_A) + \beta_X^{\top}X\right\} \tag{e1}$$

where $\lfloor t\rfloor$ denotes the time of the most recent visit before time t, $\bar{a}_{\lfloor t\rfloor} = \{a_0, a_1, \dots, a_{\lfloor t\rfloor}\}$ denotes treatment history up to time $\lfloor t\rfloor$, and $g(\bar{a}_{\lfloor t\rfloor};\beta_A)$ denotes a function of that history. The MSM could be extended to incorporate interactions between treatment and components of X that are known or suspected treatment effect modifiers.

When there is time-dependent confounding, as in main Figure 1, fitting the MSM directly using the observed development data will result in biased estimates of the parameters. However, the MSM can be estimated using IPTW under the assumptions of conditional sequential exchangeability (no unmeasured confounding), consistency, and positivity. These assumptions are as stated in the main text section on *Artificial censoring and inverse probability weighting*. The weight for a given individual at time t is the inverse of the conditional probability of having had their observed treatment pattern up to time t given their past treatment status and time-dependent covariate history. Under the assumed data structure illustrated, the covariate history required to control confounding is $\bar{L}_t$, and the weights are

$$\prod_{s=0}^{\lfloor t\rfloor} \frac{1}{\Pr(A_s = a_s|\bar{A}_{s-1} = \bar{a}_{s-1}, \bar{L}_s)}. \tag{e2}$$

Stabilized weights can be used, for example of the form

$$\prod_{s=0}^{\lfloor t\rfloor} \frac{\Pr(A_s = a_s|\bar{A}_{s-1} = \bar{a}_{s-1}, X)}{\Pr(A_s = a_s|\bar{A}_{s-1} = \bar{a}_{s-1}, \bar{L}_s)}. \tag{e3}$$

Any baseline variables that are confounders and that are conditioned on in the model in the numerator of the stabilized weights must be included in the MSM. In this case, as the MSM includes X, the model in the numerator of the stabilized weights can include all or a subset of the variables in X. A detailed discussion of weight stabilisation is given in Hernan and Robins (Section 12.3).[2] The weights are typically estimated using logistic regressions for treatment status at each visit time, or a pooled logistic regression across time points.

The MSM in (e1) can then be fitted using the time-updated weights. Some applications of the MSM-IPTW approach have focused on a discrete time setting and use pooled logistic regression models fitted over series of time intervals, which is asymptotically equivalent to a Cox regression when the discrete time-periods get small. Risk under the treatment strategy $\underline{a}_0$, as defined in main text equation (1), can then be estimated using the relation

$$R^{\underline{a}_0}(\tau|X;\boldsymbol{\beta}) = 1 - \exp\left\{-\int_0^{\tau} h_{T^{\underline{a}_0}}(u \mid X;\boldsymbol{\beta})du\right\}. \tag{e4}$$

Development using the cloning-censoring-weighting approach

The cloning-censoring-weighting approach is a closely related alternative to MSM-IPTW that focuses on estimation of risks under a restricted set of treatment strategies of interest, whereas the MSM in equation (e1) enables estimation under any longitudinal treatment strategy. The first

step in this approach ('cloning') is to create a copy of the development dataset for each strategy of interest. In the 'censoring' step, the dataset corresponding to a given treatment strategy $\underline{a}_0$ is modified such that each individual's follow-up is used for the duration for which their treatment status is consistent with the strategy $\underline{a}_0$, by censoring people when their observed treatment status deviates from that strategy. For example, if we were interested in the *never treated* ($\underline{a}_0 = \mathbf{0}$) and *always treated* ($\underline{a}_0 = \mathbf{1}$) strategies we would create two copies of the data. In the *never treated* dataset individuals would be censored at the visit time at which they initiate treatment ($A_k = 1$), if that occurs, and individuals with $A_0 = 1$ would have zero follow-up. Similarly, in the *always treated* dataset individuals would be censored at the visit time at which they stop taking treatment ($A_k = 0$), meaning that individuals with $A_0 = 0$ would have zero follow-up. Because time-dependent covariates are associated with changes in treatment status and with the outcome, the 'artificial' censoring is informative, and this is addressed in the analysis by using inverse probability of artificial censoring weighting (IPACW). In the dataset for treatment strategy $\underline{a}_0$, the IPACW at time t for an individual who has not yet had the event or been artificially censored is the inverse of the probability of having sustained treatment strategy $\underline{a}_0$ up to time t, meaning that the weights for individuals not artificially censored are the same as in (e2) or (e3). To obtain estimates of conditional risks a model for the conditional hazard $h_{T^{\underline{a}_0}}(t|X)$ could be fitted for each treatment strategy of interest, i.e. separately for each of the modified datasets. Alternatively, combined models could be fitted. For example, if we were interested in the *never treated* and *always treated* strategies the MSM could be a stratified Cox model of the form

$$h_{T^{\underline{a}_0=a}}(t|X;\boldsymbol{\beta}) = h_{0a}(t)\exp\left\{\beta_X^\top X\right\}, \qquad a = 0, 1. \tag{e5}$$

Risks under the treatment strategies of interest, as defined in main text equation (1), can then be estimated using the relation in (e4).

We emphasise that the weights used in the development step for an interventional prediction model are different from those used in the validation step, as treatment assignment may be different in the development and validation datasets. Even if the data follow the same structure, our validation approach uses unstabilised weights.

eAppendix 2 Counterfactual cumulative dynamic AUCt

Several versions of AUCt have been proposed.[3] We focus on the cumulative/dynamic AUCt, which measures discrimination *at* a particular time point t. A corresponding c-index, which summarizes discrimination over a range of follow up times *up to* a time horizon τ, was discussed in the section on *Counterfactual discrimination* in main text.

More specifically, the cumulative/dynamic AUCt assesses concordance of predictions for pairs comprising cumulative cases (subjects with $T \leq t$), and dynamic controls (subjects with $T > t$).[3] Under treatment strategy $\underline{a}_0$ the cumulative/dynamic AUCt is defined as

$$AUC^{\underline{a}_0}_{C/D}(t) = \Pr(\hat{R}^{\underline{a}_0}(t|X_i) > \hat{R}^{\underline{a}_0}(t|X_j)|T_i^{\underline{a}_0} \leq t, T_j^{\underline{a}_0} > t). \tag{e6}$$

We propose the following weighted estimator for $AUC^{\underline{a}_0}_{C/D}(t)$:

$$A\hat{U}C^{\underline{a}_0}_{C/D}(t) = \frac{\sum_{i=1}^n \sum_{j=1}^n I(\hat{R}^{\underline{a}_0}(t|X_i) > \hat{R}_j^{\underline{a}_0}(t|X_j))\text{comp}^{(3)}_{\underline{a}_0,ij}(t)\hat{W}^{(3)}_{\underline{a}_0 ij}}{\sum_{i=1}^n \sum_{j=1}^n \text{comp}^{(3)}_{\underline{a}_0,ij}(t)\hat{W}^{(3)}_{\underline{a}_0,ij}} \tag{e7}$$

where $\text{comp}^{(3)}_{\underline{a}_0,ij}(t) = I(\tilde{T}_{\underline{a}_0 i} \leq t, \tilde{T}_{\underline{a}_0 j} > t, \tilde{D}_{\underline{a}_0 i} = 1)$ indicates whether the pair of subjects (i,j) is comparable at time t in $V^{\underline{a}_0}$, and $\hat{W}^{(3)}_{\underline{a}_0,ij} = \hat{G}^{-1}_{\underline{a}_0 c}(\tilde{T}_{\underline{a}_0 i}|L_i)\hat{G}^{-1}_{\underline{a}_0 c}(t|L_j)$ is the weight of the pair.

eAppendix 3 Simulation Plan

We follow the general recommendations of Morris et al.[4] on the conduct of simulation studies for evaluating statistical methods.

Aim

The aim of this simulation study is to evaluate the performance of our proposed methods for assessing predictions under interventions using longitudinal observational data. Specifically, we aim to investigate whether the proposed counterfactual performance measures (calibration, discrimination, and Brier score) give unbiased estimates of the true performance measures that would be observed if we had a perfect validation set where everyone followed the treatment strategy of interest. Our interest includes the ability of the methods to detect poor predictive performance.

Data generating mechanisms

We generate development datasets, observational validation datasets and 'perfect' validation datasets. The perfect validation datasets allow construction of the true performance measures. All datasets are generated according to the longitudinal structure illustrated in the DAG in eFigure 2, with treatment A_k and time-dependent covariate L_k that introduces time-dependent confounding being generated at five visits ($k = 0, 1, 2, 3, 4$), alongside the continuous time to event. Compared to the general DAG introduced in the main text, we do not use P in the simulation, meaning that in this case X, the conditioning set in the interventional prediction model, only contains L_0. The DAG in eFigure 2 additionally accounts for an unobserved variable U that affects L and Y, to introduce some realistic unexplained randomness in the longitudinal marker and the outcome. Note that U does not affect A, so it does not introduce confounding.

Data generation requires models for $A_k|\bar{A}_{k-1}, \bar{L}_k$, for $L_k|\bar{A}_{k-1}, \bar{L}_{k-1}, U$, and for the hazard for the event at time t conditional on the treatment and covariate history, denoted $h(t|A_{\lfloor t \rfloor}, L_{\lfloor t \rfloor}, U)$.

Three main scenarios are considered. In Scenario 1, the development and validation datasets are generated under the same model for the conditional hazard $h(t|A_{\lfloor t \rfloor}, L_{\lfloor t \rfloor})$. In Scenario 2 the development dataset has a higher baseline hazard than the validation dataset, but the form of the hazard model is otherwise the same. In Scenario 3 the development and validation datasets are generated under the same mechanism as in Scenario 1, but the predictions in the validation data are obtained using an error prone version of L_0, denoted L_0^*. Scenarios 2 and 3 mimic settings where we expect poor predictive performance.

Three additional scenarios were considered in which we assessed our method's performance under respective violation of the assumptions of positivity (Scenarios 4a-b), conditional exchangeability (Scenarios 5a-b), and correct specification of the weight model (Scenarios 6a-d).

For each scenario we consider data generating mechanisms using an additive hazards model[5] for $h(t|A_{\lfloor t \rfloor}, L_{\lfloor t \rfloor}, U)$ and using a proportional hazards model for $h(t|A_{\lfloor t \rfloor}, L_{\lfloor t \rfloor}, U)$. The data generating mechanisms are summarised in detail in eTable 1 for the additive hazards model and in eTable 2 for the proportional hazards model. The reason for considering a data generating mechanism using an additive hazards model is that it enables us to fit a correctly specified MSM

during model development for $h_{T^{\underline{a}_0}}(t|L_0)$, as the form of $h_{T^{\underline{a}_0}}(t|L_0)$ is then also an additive hazards model.[6] This enables us to assess the proposed validation methods in an 'ideal' scenario in which the development model is correctly specified and the development and validation data are generated under the same mechanism (Scenario 1). When $h(t|A_{\lfloor t \rfloor}, L_{\lfloor t \rfloor})$ is a conditional proportional hazards model, the MSM for $h_{T^{\underline{a}_0}}(t|L_0)$ is no longer a proportional hazards model and in fact is of a complex non-standard form. However, a data generating mechanism based on a proportional hazards model is more widely familiar. Including a data generating scenario using a proportional hazards model also provides one example of a situation in which the development model will be mis-specified so we would expect the risks estimated from this model to be biased estimates of the true risks under interventions in the validation data. This model mis-specification should be reflected in the performance measures.

We chose a sample size of n=3000 in each simulation run. This choice was motivated by having at least 80% power to accurately (within 5% margin of error) estimate the overall outcome proportion using the artificially censored data in each scenario.[7] We performed 1000 simulation runs.

Estimands

The estimands of interest are the counterfactual measures of predictive performance for assessment of predictions under interventions introduced in the main paper (equation (1) with $X = L_0$). We obtain estimates of risk for each individual in the observational validation dataset under the treatment strategies of interest, in our case the *always treated* and *never treated* strategies, at time horizons $\tau = 1, 2, 3, 4, 5$. Our aim is not to consider the accuracy or precision of these risk estimates, but to assess their predictive performance. To obtain 'true' values of predictive performance, we extend each generated observational validation dataset into two 'perfect' validation datasets, one for the *always treated* and one for the *never treated* strategy. These perfect validation datasets inherit the baseline values L_0 and U from the observational validation dataset. Later values $L_k, k = 1, 2, 3, 4$ and the times to event are generated assuming all patients follow the *always treated* or the *never treated* strategy. These perfect validation datasets constitute the ideal validation setting for the predictions under interventions: as all patients follow the strategy of interest, standard estimators for performance measures can be used. In the observational validation dataset we calculate the performance measures listed in eTable 3 and we compare these to the measures obtained from the perfect validation datasets.

Methods

An interventional prediction model is fitted in the development data using an MSM-IPTW analysis as outlined in eAppendix 1. The development model is used to obtain estimates of risks for each person at time horizons $\tau = 1, 2, 3, 4, 5$ in the observational validation data under the *always treated* strategy and under the *never treated* strategy. In main Scenarios 1 and 2 (and additional Scenarios 4-6) this is done using L_0 in the validation data. In Scenario 3 the predictions are obtained using an error prone version of baseline measurements L_0^*.

The counterfactual performance measures as described eTable 3 are estimated from the observational validation data following two approaches. First, we apply the subset approach as described in the *Introduction* section in the main paper. This means we apply standard predictive performance measures to the subset of patients in the observational validation data who followed the treatment strategy of interest (*always treated* or *never treated*). The subset is specific to the time horizon over which a certain performance measure is calculated. Second, we apply the proposed artificial censoring and inverse probability weighting approach to assess counterfactual performance. Note that the weights used during the validation are estimated in the validation data. As

depicted in eTables 1(a) and 2(a), the models used to generate treatment assignment A_k only depend on the latest value of $\bar{L}_k$, L_k. Accordingly, the weight models, depicted in eTables 1(c) and 2(c) only condition on this latest value L_k (accept in Scenario 6 where we introduce misspecification in the weight models).

Performance measures

For each simulation run, we will calculate the counterfactual performance measures estimated from the observational validation data and the true performance measure calculated from the perfect validation datasets. We depict the estimates graphically and tabulate the mean of their differences (i.e. the bias) and the Monte Carlo standard error of the bias.

eAppendix 4 Simulation Results

Simulation Descriptives

The marginal risk distributions averaged over the 1000 simulation runs and from the development data, observational validation data and the two perfect validation datasets for the main Scenarios 1-3 generated under the additive hazards model are shown in eFigure 3. In the *never treated* perfect validation data the average risk by time point 5 is 70% (2100 events). The corresponding marginal risk from the *always treated* perfect validation data is 62% (1866 events on average by time point 5), meaning that treating all subjects would lower the overall risk by about 8 percentage points compared to not treating any patient. In the development data (for Scenarios 1 and 3) and observational validation data (for Scenarios 1-3), on average 53% of patients started treatment at some point during follow up. The mix of treated and untreated individuals in the development (Scenarios 1 and 3) and observational validation data led to an overall risk of 66% by time point 5 (on average 1980 events). In the artificially censored validation data for the *never treated* strategy (V^0) on average 1122 events remained in the analysis and in the artificially censored validation data for the *always treated* strategy (V^1) 534 events remained.

The risk distributions for the proportional hazards based scenarios where we assumed a stronger treatment effect, a stronger effect of L and a higher percentage of patients who received treatment, are presented in eFigure 4. Under the proportional hazards model in the *never treated* perfect validation data the average risk by time point 5 is 59% (1769 events). In the corresponding *always treated* perfect validation data, the average risk by time point 5 was 24% (729 events), so 35 percentage points lower. In the development data (Scenarios 1 and 3) and observational validation data, on average 68% of patients started treatment at some point during follow up. The mix of treated and untreated individuals in the development data (Scenarios 1 and 3) and observational validation data led to an average risk of 40% by time point 5. In the artificially censored validation data for the *never treated* strategy (V^0) on average 680 events remained in the analysis and in the artificially censored validation dataset for the *always treated* strategy (V^1) 227.

Simulation results proportional hazards scenarios

Results for the scenarios in which data were generated and analysed using a proportional hazards model are presented in eTables 7, 8, 9 and 11 and eFigures 8 to 13. As explained in eAppendix 1, using the proportional hazards model during model development will lead to a mis-specified MSM based on a Cox proportional hazards model and this is reflected in the true calibration curve not lying perfectly on the diagonal in Scenario 1. Results for the proportional hazards model confirm the unbiasedness of the proposed estimators when the necessary assumptions are met (Scenarios

1-3) and show somewhat stronger biases (compared to the additive hazards based data generation) for the scenarios with deliberately introduced violations of causal assumptions (Scenarios 4-6). This is attributable to the stronger treatment effect and stronger effects of L in these proportional hazards based scenarios. The bias of the proposed estimators was smaller than that of the naive subset method in 52/64 (81%) of times in the proportional hazards based scenarios (eTable 11).

Discussion of the size and direction of bias when using the subset method

Bias in the estimates using the subset method were more pronounced and sometimes in opposite directions for the *never treated* strategy compared to the *always treated* strategy. Here we explain the mechanisms behind this. Under the *never treated* strategy, the subset is obtained by excluding subjects if they start treatment at any time point during the follow-up time of interest, meaning that we have 'selection based on the future'. Notably, subjects who experience the event can no longer be excluded after that, introducing a type of 'immortal time bias'. This leads to an over representation of events in the resulting subset under the *never treated* strategy and explains the overestimation of observed outcome proportions by the subset method under that strategy. For the *always treated* strategy, selection into the subset is based only on the first visit. Patients are excluded if they do not start treatment directly, but remain in the analysis after that because under our data generating mechanism individuals always continue treatment after it is initiated. The exclusion decision is made at time zero before any events are recorded, meaning that there is no 'immortal time' in the *always treated* subset. Selection into the subset in the *always treated* strategy is however not completely at random. Due to the positive relation between L_0 and A_0, the subjects who receive treatment at time 0 and who thus stay in the subset under the *always treated* strategy on average have higher and more homogeneous underlying risk compared to the counterfactual *always treated* data (eFigure 2). With a more homogeneous risk distribution in the subset under the *always treated* strategy compared to the perfect validation data under the *always treated* strategy, one can expect measures of discrimination to decrease.[8–10] This explains the underestimation of discrimination indices seen with the subset approach for this strategy. Discrimination indices for the *never treated* strategy are overestimated by the subset method. This would be consistent with a more heterogeneous underlying risk distribution in the subset used under the *never treated* strategy compared to the risk distribution in the *never treated* perfect validation data. Low risk subjects, who corresponds to low L values, are at low risk of treatment initiation and are thus more often retained in the subset for the *never treated* strategy. High risk subjects, corresponding to high L values, may be excluded from this subset if they start treatment but we also noted that if they experience an event they are retained in the subset for the *never treated* strategy (eFigure 2). Apparently, these two processes in effect lead to a more heterogeneous risk distribution explaining the overestimation of discrimination indices by the subset method under the *never treated* strategy.

eAppendix 5 Liver transplant application

Data overview

This study used data from the Scientific Registry of Transplant Recipients (SRTR). The SRTR data system includes data on all donors, wait-listed candidates, and transplant recipients in the US, submitted by the members of the Organ Procurement and Transplantation Network (OPTN). The Health Resources and Services Administration (HRSA), U.S. Department of Health and Human Services provides oversight to the activities of the OPTN and SRTR contractors. The data reported here have been supplied by the Hennepin Healthcare Research Institute (HHRI) as the contractor

for the Scientific Registry of Transplant Recipients (SRTR). The interpretation and reporting of these data are the responsibility of the author(s) and in no way should be seen as an official policy of or interpretation by the SRTR or the U.S. Government.

For this study we restricted to individuals who joined the liver transplant waitlist between 1 January 2014 and 30 April 2019, due to a change in the organ allocation policy in 2019. Administrative censoring was applied at the earlier of 3 years after joining the waitlist or at 30 April 2019. We excluded people with missing information on time-fixed variables (mostly underlying disease group was missing). We also excluded individuals with missing data for time dependent variables, as the number was very small. The data include date of receiving a transplant, date of death (pre- or post-transplant), and date of and reason for removal from the waitlist. The reason for being removed from the waitlist can be due to worsening health status, due to improvement in health status or for "other" reasons. We consider a composite outcome of death or removal from the transplant waitlist due to worsening health status. For convenience below we refer to removal due to improvement in health status or for "other" reasons collectively as improvement-based-removal.

Before exclusions due to missing data, the data included 50552 individuals. We excluded 7108 (14.1%) individuals with missing information on their disease group. A further 242 individuals were excluded because they had missing data on baseline covariates of region, diabetes status or BMI. Lastly we excluded 12 people who had any missing values in time-dependent variables at any measurement time. Missingness occurred only for INR, ascites, encephalopathy, and Child Pugh Score grade. After exclusions, the data included 43190 individuals.

Data set-up

We let s denote time in days since joining the waitlist. The two interventions under which we aim to make predictions are: (1) receiving a liver transplant at time s; (2) not receiving a transplant at time s or in the future. The two interventions could be applied at any time s that an individual is on the waitlist pre-transplant. We therefore wish to be able to make predictions from any time s from the time of joining the waitlist and we consider $0 \leq s < 1096$ (where 1096 days is approximately 3 years).

To enable development of predictions under the two interventions at any time s after joining the waitlist we create two new datasets. We let D_1 denote a dataset formed of individuals who receive a transplant within 3 years of joining the waitlist, followed-up from the time of transplant onwards. We also created datasets starting at a series of landmark times from the time of joining the waitlist. We used landmark times at 90-day intervals up to 3 years, giving 13 landmark datasets starting at times $s = 0, 90, 180, \dots, 900, 990, 1080$. The dataset starting at landmark time s includes individuals who remain on the waitlist at time s - that is, people who have not had a transplant up to time s, who have not had the composite event up to time s, who have not been removed from the waitlist due to improvement or for "other" reasons up to time s (improvement-based-removal), and who have not been administratively censored up to time s. The landmark datasets are combined into a single stacked dataset, denoted D_0. Individuals can contribute to more than one landmark dataset in D_0, and individuals who have a transplant within 3 years of joining the waitlist contribute to both D_1 and D_0. In dataset D_1 time zero is the day of transplant. In dataset D_0 time zero is the landmark time. We let D denote datasets D_1 and D_0 combined. When an individual appears more than once in D we treat the different contributions as separate observations for the analysis, referred to as 'person-landmark observations'. To facilitate this we generate a new ID-number "`id.`s", which refers to a person-landmark observation, where `id` denotes the original anonymised unique identifier, and s denotes the landmark time (for D_0) or the

time of transplant (for D_1).

The combined dataset D was divided randomly into a 70% sample used for model development and a 30% sample used for the validation, with the sampling being performed on the basis of person-landmark observations using the unique identifiers "`id.`s". We let $D^{dev} = \{D_0^{dev}, D_1^{dev}\}$ denote the development data and $D^{val} = \{D_0^{val}, D_1^{val}\}$ denote the validation data.

Dataset D_1^{dev} includes 16605 individuals who had a transplant within 3 years of joining the waitlist. Among these individuals there were 1748 composite events within 3 years of post-transplant follow-up. The remaining individuals were censored, either because they were still alive after 3 years of follow-up or because of end of follow-up at 30 April 2019. Dataset D_0^{dev} includes 100192 person-landmark observations, which includes some individuals counted in multiple landmark datasets (there are 37210 unique individuals in D_0^{dev}). Out of the 100192 person-landmark observations, there were 16717 composite events (death without a transplant or removals from the waitlist due to worsening health status) within 3 years of the landmark time, 40857 had a transplant within 3 years of the landmark, 18137 had improvement-based-removal, and the remaining 24481 person-landmark observations were censored (pre-transplant) because they were still alive after 3 years of follow-up or because of end of follow-up at 30 April 2019.

Dataset D_1^{val} includes 7146 individuals who had a transplant within 3 years of joining the waitlist. Among these individuals there were 777 composite events within 3 years of post-transplant follow-up. The remaining individuals were censored, either because they were still alive after 3 years of follow-up or because of end of follow-up at 30 April 2019. Dataset D_0^{val} includes 42820 person-landmark observations, which includes some people counted in multiple landmark datasets (there are 24228 unique individuals in D_0^{val}). Out of the 42820 person-landmark observations, there were 7221 composite events (death without a transplant or removals from the waitlist due to worsening health status) within 3 years of the landmark time, 17293 had a transplant within 3 years of the landmark, 7883 had improvement-based-removal, and the remaining 10423 person-landmark observations were censored (pre-transplant) because they were still alive after 3 years of follow-up or because of end of follow-up at 30 April 2019.

The analysis makes use of time-fixed covariates Z and time-dependent covariates, with L_s denoting the time dependent covariates as measured at time s after joining the waitlist. The following time-fixed covariates were included in Z: sex, ethnicity (Asian, Black, White, Other), blood group (A, B, AB, O), region of residence (11 regions), disease group (Alcoholic cirrhosis, Hepititis B virus (HBV) cirrhosis, Hepititis C virus (HCV) cirrhosis, Cryptogenic, Hepatocellular carcinoma (HCC), Non-alcohol related steatohepatitis (NASH), Primary biliary cirrhosis (PBC), Primary sclerosing cholangitis (PSC)), diabetes, BMI. The following time-dependent variables were included in L_s: age, chronic kidney disease, dialysis (if the patient had dialysis within the week prior to the serum creatinine test), components of the MELD-NA score (creatinine, bilirubin, INR, sodium), albumin, ascites (Absent, Slight, Moderate), encephalopathy (None, 1-2, 3-4), Child Pugh Score grade (A, B, C), number of tumours (0, 1, 2 or more), whether the person has exception points (yes, no), whether the person has exception points due to HCC (yes, no). Further details about how these are used in the analysis are provided below.

Characteristics of person-landmark observations in the development and validation datasets at time of joining the waiting list ($s = 0$) are summarised in eTables 12 and 13, and summaries of numbers of events and censorings in the validation datasets are given in eTable 14.

Development of interventional prediction models

The data set-up described above and our corresponding analysis are related to approaches taken in earlier studies that have investigated the benefits of organ transplant, though earlier studies have focused on estimating average (or conditional average) effects of transplant rather than on predictions under interventions. Our approach is closely related to that of Gong and Schaubel,[11] who estimated the effect of liver transplant in the transplanted, stratified by MELD score. Their data set-up differs from ours in that their landmark times were defined in terms of calendar time rather than in terms of time since joining the waitlist. Strohmeier et al.[12] used a related approach, but they set their landmark times at each transplant time (relative to moment of joining the waitlist), and their approach is in turn related to the sequential stratification approach of Schaubel et al.[13,14] In this application, our strategies of interest for prediction under interventions do not require assumptions about resource constraint that is faced in organ transplantation. However, in future work other treatment strategies of interest for prediction under interventions may require consideration of the resource constraint, for example the strategy of 'waiting until the next donor organ becomes available'.

Prediction under transplant

We developed separate models for prediction under the two interventions. Dataset D_1^{dev} was used to develop a prediction model under the intervention of receiving a liver transplant at time s ($0 \leq s < 1096$). A Cox model was fitted, with time of transplant as time zero. The model includes as predictors time-fixed variables Z and time-dependent variables L_s as measured just prior to transplant, i.e. $X = \{Z, L_s\}$. All continuous covariates were modelled using restricted cubic splines with 3 knots. The model also included s (time of transplant) as a covariate, which was modelled using a restricted cubic splines with four knots placed at s corresponding to $180, 360, 720, 900$ days. Time-dependent variables were not recorded daily, and we used the last observation carried forward for the values of L_s.

Prediction under no transplant

Dataset D_0^{dev} was used to develop a prediction model under the intervention of not receiving a liver transplant at time s or in the future ($0 \leq s < 1096$). All individuals in D_0^{dev} have not received a transplant before their landmark time, but many receive a transplant after the landmark time. We use the censoring-and-weighting approach to obtain predictions under the intervention of interest - that is, not not receiving a transplant now or in the future. In the censoring step, individuals in D_0^{dev} were censored at the time of receiving a transplant. The resulting modified dataset is denoted $D_0^{dev,cens}$. The artificial censoring at transplant is dependent on time-dependent characteristics of the individual. This is addressed in the analysis using time-dependent inverse probability of artificial censoring weights (IPACW). In addition to the artificial censoring there is administrative censoring due to the end of follow-up at 3 years or at 30 April 2019, and censoring because of improvement-based-removal. The administrative censoring is assumed to be uninformative. However, improvement-based-removal is likely to depend on time-updated individual characteristics that are also associated with the persons subsequent hazard for mortality. We use a second set of weights to address this, referred to as inverse probability of removal weights (IPRW). Once an individual is removed from the waitlist they are assumed not to return.

For estimating the IPACW and IPRW we divided each individual's follow-up into periods of length 30 days, starting from the landmark time, enabling use of pooled logistic regression to estimate the weights. Time-dependent covariates are updated at the start of each 30 day period. We let A_{s+30k} denote the transplant status at the start of the kth 30-day period following landmark time

s, with $A_s = 0$ denoting transplant status at the landmark time, L_{s+30k} denote the values of time-dependent covariates at the start of period k after landmark time s, L_s denote the values of time-dependent covariates at the landmark time, and Z denote time-fixed covariates. We let $Q_{s+30k} = 1$ denote that an individual is removed from the waitlist at the end of period k after the landmark, and $Q_{s+30k} = 0$ otherwise. Time-dependent variables were not recorded daily, and we used the last observation carried forward for the values of L_s.

For landmark time s, the stabilised IPACW in period k after the landmark is

$$\text{IPACW}(s,k) = \frac{\prod_{j=1}^{k} \Pr(A_{s+30j} = 0|\bar{A}_{s+30j-30} = 0, Q_{s+30j-30} = 0, L_s, Z)}{\prod_{j=1}^{k} \Pr(A_{s+30j} = 0|\bar{A}_{s+30j-30} = 0, Q_{s+30j-30} = 0, L_{s+30j}, Z)}, \quad \text{(e8)}$$

and the stabilised IPRW in period k after the landmark is

$$\text{IPRW}(s,k) = \frac{\prod_{j=1}^{k} \Pr(Q_{s+30j-30} = 0|\bar{A}_{s+30j-30} = 0, \bar{Q}_{s+30j-60} = 0, L_s, Z)}{\prod_{j=1}^{k} \Pr(Q_{s+30j-30} = 0|\bar{A}_{s+30j-30} = 0, \bar{Q}_{s+30j-60} = 0, L_{s+30j-30}, Z)}. \quad \text{(e9)}$$

The total weight for a given individual in period k after the landmark is the product $\text{IPACW}(s,k) \times \text{IPRW}(s,k)$. In period $k = 0$ the weight is equal to 1 for all individuals in D_0^{dev}.

The probabilities used in the weights were estimated using logistic regression. The covariates included were the same as those listed above, with the exception that we included the MELD-NA score in L_s instead of the individual components of the MELD-NA score (creatinine, bilirubin, INR, sodium). All continuous covariates were modelled using restricted cubic splines with 3 knots. The models also included current time as a covariate, which was modelled using a restricted cubic splines with four knots placed at $30k$ corresponding to $180, 360, 720, 900$ days.

A Cox model was fitted using the dataset $D_0^{dev,cens}$, with landmark time as time zero. The model was fitted using the time-dependent weights ($\text{IPACW}(s,k) \times \text{IPRW}(s,k)$: updated every 30 days) and using predictors Z and L_s as measured at the landmark time. All continuous covariates were modelled using restricted cubic splines with 3 knots. The models also included s (the landmark time) as a covariate, which was modelled using a restricted cubic splines with four knots placed at s corresponding to $180, 360, 720, 900$ days.

Validation

The validation dataset $D^{val} = \{D_0^{val}, D_1^{val}\}$ includes individuals for whom a prediction under the two interventions could be made at a range of times s. We applied the proposed approach of artificial censoring to the dataset D^{val} to generate validation datasets V^1 and V^0 mimicking the two strategies. To mimic the strategy of receiving a transplant at a time s in V^1, person-landmark observations in D_0^{val} are artificially censored immediately at the landmark time, whereas follow-up on individuals in D_1^{val} is retained. To mimic the strategy of not receiving a transplant at a time s or in the future in V^0, individuals in D_1^{val} are artificially censored immediately at time zero (the time of transplant), and person-landmark observations in D_0^{val} are artificially censored at the time of transplant, if they received a transplant.

Created in this way, V^1 contains individuals artificially censored at time zero (the landmark time) but there is no artificial censoring at later times. We therefore estimate time-fixed IPACW, which are the inverse of the probability of receiving a transplant at time 0. The weight for an individual in V^1 transplanted at time s is $G_1^{-1}(t|L,Z) = 1/\Pr(A_s = 1|\bar{A}_{s-1} = 0, L_s, Z)$, where t denotes follow-up time starting from transplant. These weights are the same at all times t.

In V^0, individuals have been artificially censored due to transplant at a range of times. We therefore require time-dependent IPACW. Censoring due to improvement-based-removal from the waitlist also needs to be accounted for. The IPACW and IPRW were estimated using a similar approach as in the development step, after dividing each individual's follow-up into periods of length 30 days, albeit using unstabilised rather than stabilised weights. For landmark time s the total weight at times $30k \leq t < 30(k+1)$ after the landmark time is

$$G_0^{-1}(t|L,Z) = \frac{1}{\prod_{j=1}^{k} \Pr(A_{s+30j} = 0|\bar{A}_{s+30j-30} = 0, Q_{s+30j-30} = 0, L_{s+30j}, Z)} \times \frac{1}{\prod_{j=1}^{k} \Pr(Q_{s+30j-30} = 0|\bar{A}_{s+30j-30} = 0, \bar{Q}_{s+30j-60} = 0, L_{s+30j-30}, Z)}. \quad \text{(e10)}$$

Unlike in the development step, in the validation data the weights are not equal to 1 in period $k = 0$ (i.e. at follow-up times $0 \leq t < 30$), because the weights also account for censoring of individuals at time 0 if they are transplanted at that time. In particular the IPACW weights (first term in $G_0^{-1}(t|L,Z)$) are not equal to 1 in period $k = 0$, whereas the IPRW (second term in $G_0^{-1}(t|L,Z)$) are still equal to 1 in period $k = 0$.

In addition to the artificial censoring and the censoring due to improvement-based-removal, there is administrative censoring due to the end of follow-up at 3 years or at 30 April 2019. This additional standard censoring needs to be addressed in some measures of counterfactual predictive performance using a further set of weights, $G_c^{-1}(t)$, as described in the main manuscript. We used the Kaplan-Meier estimator on the observed validation data D^{val} (so before artificial censoring was applied) to estimate the probability of remaining administratively uncensored up to any given time, where time is measured relative to the time of transplant (for individuals in D_1^{val}) or relative to the landmark time (for person-landmark observations in D_0^{val}). The weights $G_c^{-1}(t)$ are given by the inverse of these probabilities.

The models developed to enable predictions under the two intervention strategies of interest were used to obtain estimated risks under both strategies for each person-landmark observation in D^{val} using their characteristics at time zero, which is the time of transplant for individuals in D_1^{val} and the landmark time for person-landmark observations in D_0^{val}. After creating the validation datasets V^1 and V^0 and estimating the weights $G_a^{-1}(t|L,Z)$ and $G_c^{-1}(t)$ we obtained estimates of the measures of counterfactual predictive performance using the methods described in the main text.

We also applied the subset approach. For this, the predictions for each person-landmark observation in D^{val} under the intervention strategy of receiving a transplant now were evaluated on the subset of individuals who received a transplant based on their post-transplant follow-up, i.e. on D_1^{val}. The predictions for each person-landmark observation in D^{val} under the intervention strategy of not receiving a transplant now or in the future were evaluated on the subset of individuals who never received a transplant, from their landmark times onwards, i.e. on the subset of D_0^{val} that never received a transplant. Some individuals in the subset of D_0^{val} that never received a transplant are censored due to improvement-based removal from the waitlist. In the subset validation approach this censoring was treated in the same way as administrative censoring.

References

[1] D.R. Cox. Regression models and life-tables. *Journal of the Royal Statistical Society (Series B)*, 34:187–220, 1972.

[2] M.A. Hernán and J.M. Robins. *Causal Inference: What If.* Boca Raton: Chapman & Hall/CRC, 2020.

[3] P.J. Heagerty and Y. Zheng. Survival model predictive accuracy and ROC curves. *Biometrics*, 61(1):92–105, 2005.

[4] T.M. Morris, I.R. White, and M.J. Crowther. Using simulation studies to evaluate statistical methods. *Statistics in Medicine*, 38:2074–2102, 2019.

[5] O.O. Aalen. A linear regression model for the analysis of life times. *Statistics in Medicine*, 8:907–925, 1989.

[6] R.H. Keogh, S.R. Seaman, J.M. Gran, and S. Vansteelandt. Simulating longitudinal data from marginal structural models using the additive hazard model. *Biometrical Journal*, 63:1526–1541, 2021.

[7] R.D. Riley, J. Ensor, K.I.E. Snell, F.E. Harrell, G.P. Martin, J.B. Reitsma, K.G.M. Moons, G. Collins, and M. van Smeden. Calculating the sample size required for developing a clinical prediction model. *BMJ*, page m441, 2020.

[8] G.A. Diamond. What price perfection? Calibration and discrimination of clinical prediction models. *Journal of Clinical Epidemiology*, 45(1):85–89, 1992.

[9] M. H. Gail. On criteria for evaluating models of absolute risk. *Biostatistics*, 6(2):227–239, 2005.

[10] R. Pajouheshnia, L.M. Peelen, K.G.M. Moons, J.B. Reitsma, and R.H.H. Groenwold. Accounting for treatment use when validating a prognostic model: a simulation study. *BMC Medical Research Methodology*, 17(1):103, 2017.

[11] Q. Gong and D.E. Schaubel. Estimating the average treatment effect on survival based on observational data and using partly conditional modeling. *Biometrics*, 73(1):134–144, 2017.

[12] S. Strohmaier, C. Wallisch, M. Kammer, A. Geroldinger, G. Heinze, R. Oberbauer, and M. Haller. Survival benefit of first single-organ deceased donor kidney transplantation compared with long-term dialysis across ages in transplant-eligible patients with kidney failure. *JAMA Netw Open*, 5(10):e2234971, 2022.

[13] D. Schaubel, R. Wolfe, and F. Port. A sequential stratification method for estimating the effect of a time-dependent experimental treatment in observational studies. *Biometrics*, 62:910–917, 2006.

[14] D. Schaubel, R. Wolfe, C. Sima, and R.M. Merion. Estimating the effect of a time-dependent treatment by levels of an internal time-dependent covariate: application to the contrast between liver wait-list and posttransplant mortality. *J Am Stat Assoc*, 104:49–59, 2009.

eTable 1: Simulation study: Data generating mechanisms and weight specifications, additive hazards model

(a) Models for A_k and L_k: same in development and validation data unless stated otherwise, where $N(\mu, \sigma^2)$ denotes a normal distribution with mean μ and variance σ^2.

All scenarios	$U \sim N(0, 2^2)$
	$L_0 \sim N(10 + U, 4^2)$
	$\text{logit} \Pr(A_0 = 1 \mid L_0) = -2 + 0.1 L_0$
	$L_k \sim N(0.8 L_{k-1} - A_{k-1} + 0.1k + U, 4^2)$ $(k \geq 1)$
	$A_k = 1$ if $A_{k-1} = 1$, $(k \geq 1)$
	$\text{logit} \Pr(A_k = 1 \mid \bar{A}_{k-1}, \bar{L}_k) = \gamma_0 + \gamma_L L_k$ if $A_{k-1} = 0$, $(k \geq 1)$
	with
	$\gamma_0 = -2$ and $\gamma_L = 0.1$
Scenario 3	measurement error in risk calculation: $L_0^* = L_0 + \epsilon, \quad \epsilon \sim N(0, 16)$
Scenario 4a	positivity violation validation data: $\gamma_0 = -0.25$
Scenario 4b	positivity violation validation data: $\gamma_0 = -0.75$
Scenario 6c	quadratic term in model for A_k validation data:
	$\text{logit} \Pr(A_0 = 1 \mid L_0) = -1 + 0.01 L_0 + 0.01 L_0^2$
	$\text{logit} \Pr(A_k = 1 \mid \bar{A}_{k-1}, \bar{L}_k) = -1 + 0.01 L_k + 0.01 L_k^2$ if $A_{k-1} = 0$, $(k \geq 1)$

(b) Models for the conditional hazard $h(k \mid \bar{A}_{\lfloor k \rfloor}, \bar{L}_{\lfloor k \rfloor}, U)$

	Development data	Validation data
Scenario 1	$\alpha_0 + \alpha_A A_{\lfloor k \rfloor} + \alpha_L(1 - 0.2(k-1)) L_{\lfloor k \rfloor} + \alpha_U U$	Same as development data
	with	
	$\alpha_0 = 0.2, \alpha_A = -0.04, \alpha_L = 0.01, \alpha_U = 0.01$	
Scenario 2	As in Scenario 1 but with $\alpha_0 = 0.3$	As in Scenario 1
Scenarios 3-6	As in Scenario 1	As in Scenario 1

(c) Weight models for $A_k, A_{k-1} = 0$

Scenarios 1-4	correctly specified	$\text{logit} \Pr(A_k = 1 \mid \bar{A}_{k-1}, \bar{L}_k)$	$\sim$	$1 + L_k$
Scenario 5a	exchangeability violation		$\sim$	1
Scenario 5b	exchangeability violation		$\sim$	$1 + L_0$
Scenario 6a	misspecified		$\sim$	$1 + \log(L_k + 20)$
Scenario 6b	misspecified		$\sim$	$1 + L_k^2$
Scenario 6c	misspecified		$\sim$	$1 + L_k$
Scenario 6d	misspecified	$\text{cauchit} \Pr(A_k = 1 \mid \bar{A}_{k-1}, \bar{L}_k)$	$\sim$	$1 + L_k$

eTable 2: Simulation study: Data generating mechanisms Cox model

(a) Models for A_k and L_k: same in development and validation data unless stated otherwise, where $N(\mu, \sigma^2)$ denotes a normal distribution with mean μ and variance σ^2.

All scenarios	$U \sim N(0, 0.1^2)$
	$L_0 \sim N(U, 1)$
	$\text{logit}\,\Pr(A_0 = 1 \mid L_0) = \gamma_0 + \gamma_L L_0$
	$L_k \sim N(0.8L_{k-1} - A_{k-1} + 0.1k + U, 1^2)$ $(k \geq 1)$
	$A_k = 1$ if $A_{k-1} = 1$, $(k \geq 1)$
	$\text{logit}\,\Pr(A_k = 1 \mid \bar{A}_{k-1}, \bar{L}_k) = \gamma_0 + \gamma_L L_k$ if $A_{k-1} = 0$, $(k \geq 1)$
	with
	$\gamma_0 = -1, \gamma_L = 0.5$
Scenario 3	measurement error in risk calculation: $L_0^* = L_0 + \epsilon, \quad \epsilon \sim N(0, 1)$
Scenario 4a	positivity violation validation data: $\gamma_0 = 0.5$
Scenario 4b	positivity violation validation data: $\gamma_0 = 0$
Scenario 6c	quadratic term in model for A_k validation data:
	$\text{logit}\,\Pr(A_0 = 1 \mid L_0) = -1 + 0.5L_0 + 0.25L_0^2$
	$\text{logit}\,\Pr(A_k = 1 \mid \bar{A}_{k-1}, \bar{L}_k) = -1 + 0.5L_k + 0.25L_k^2$ if $A_{k-1} = 0$, $(k \geq 1)$

(b) Models for the log of the conditional hazard: $\log(h(k \mid \bar{A}_{\lfloor k \rfloor}, \bar{L}_{\lfloor k \rfloor}))$

	Development data	Validation data
Scenario 1	$\alpha_0 + \alpha_A A_{\lfloor k \rfloor} + \alpha_L L_{\lfloor k \rfloor} + \alpha_U U$	Same as development data
	with	
	$\alpha_0 = -2, \alpha_A = -0.5, \alpha_L = 0.5, \alpha_U = 0.5$	
Scenario 2	As in Scenario 1 but with $\alpha_0 = -1$	As in Scenario 1
Scenarios 3-6	As in Scenario 1	As in Scenario 1

(c) Weight models for $A_k, A_{k-1} = 0$

Scenarios 1-4	correctly specified	$\text{logit}\,\Pr(A_k = 1 \mid \bar{A}_{k-1}, \bar{L}_k)$	$\sim$	$1 + L_k$
Scenario 5a	exchangeability violation		$\sim$	1
Scenario 5b	exchangeability violation		$\sim$	$1 + L_0$
Scenario 6a	misspecified		$\sim$	$1 + \log(L_k + 20)$
Scenario 6b	misspecified		$\sim$	$1 + L_k^2$
Scenario 6c	misspecified		$\sim$	$1 + L_k$
Scenario 6d	misspecified	$\text{cauchit}\,\Pr(A_k = 1 \mid \bar{A}_{k-1}, \bar{L}_k)$	$\sim$	$1 + L_k$

eTable 3: Summary of counterfactual performance measures for assessment of predictions under interventions

Description	Notation
**Calibration**	
Mean estimated risk by time τ under strategy $\underline{a}_0$	$\bar{R}^{\underline{a}_0}(\tau)$, $a = \mathbf{0}, \mathbf{1}$
Counterfactual outcome proportion by time τ under strategy $\underline{a}_0$	$\bar{R}^{\underline{a}_0}_{\text{Obs}}(\tau)$, $a = \mathbf{0}, \mathbf{1}$
Ratio of "observed" versus "expected" risk by time τ under strategy $\underline{a}_0$	$\bar{R}^{\underline{a}_0}_{\text{Obs}}(\tau)/\bar{R}^{\underline{a}_0}(\tau)$, $a = \mathbf{0}, \mathbf{1}$
Mean estimated risks $\bar{R}^{\underline{a}_0}(\tau)$ within tenths of the estimated risks by time τ under strategy $\underline{a}_0$	-
Counterfactual outcome proportions within tenths of the estimated risks by time τ under strategy $\underline{a}_0$	-
**Discrimination**	
C-index truncated at time τ under strategy $\underline{a}_0$	$C^{\underline{a}_0}(\tau)$, $a = \mathbf{0}, \mathbf{1}$
Cumulative/dynamic area under the receiver operating characteristic curve at time t under strategy $\underline{a}_0$	$AUC^{\underline{a}_0}_{C/D}(t)$, $a = \mathbf{0}, \mathbf{1}$
**Brier score**	
Brier score at time t under strategy $\underline{a}_0$	$BS^{\underline{a}_0}(t)$, $a = \mathbf{0}, \mathbf{1}$
Scaled Brier score at time t under strategy $\underline{a}_0$	$1 - BS^{\underline{a}_0}(t)/BS^{\underline{a}_0}_0(t)$, $a = \mathbf{0}, \mathbf{1}$

eTable 4: Simulation results, Scenario 1, additive hazards model. Performance measures were obtained from the perfect validation data (true) and estimated from the observational validation data using the subset approach (subset) and using the proposed artificial censoring + inverse probability weighting estimators for assessing counterfactual performance (counterfactual). Results are averaged over 1000 simulation runs of validation datasets of size 3000.

	never treated			always treated		
	true	subset	counterfactual	true	subset	counterfactual
**Calibration: observed/expected ratio based on risk by time 5**						
mean	1.002	1.145	1.002	1.003	1.003	1.003
bias (SE)		0.143 (0.001)	-0.000 (0.001)		0.000 (0.001)	0.000 (0.001)
**Discrimination: C-index up to time 5**						
mean	0.546	0.578	0.547	0.555	0.552	0.556
bias (SE)		0.031 (0.000)	0.000 (0.000)		-0.003 (0.000)	0.001 (0.000)
**Discrimination: AUCt at time 5**						
mean	0.571	0.629	0.572	0.580	0.578	0.582
bias (SE)		0.058 (0.001)	0.000 (0.001)		-0.003 (0.001)	0.001 (0.001)
**Prediction error: scaled Brier score (%) at time 5**						
mean	1.201	-3.036	1.177	1.723	1.474	1.655
bias (SE)		-4.237 (0.076)	-0.024 (0.033)		-0.250 (0.033)	-0.068 (0.042)

AUCt: cumulative/dynamic area under the receiver operating characteristic curve.

eTable 5: Simulation results, Scenario 2, additive hazards model. Performance measures were obtained from the perfect validation data (true) and estimated from the observational validation data using the subset approach (subset) and using the proposed artificial censoring + inverse probability weighting estimators for assessing counterfactual performance (counterfactual). Results are averaged over 1000 simulation runs of validation datasets of size 3000.

	never treated			always treated		
	true	subset	counterfactual	true	subset	counterfactual
**Calibration: observed/expected ratio based on risk by time 5**						
mean	0.858	0.973	0.857	0.809	0.822	0.809
bias (SE)		0.115 (0.001)	-0.000 (0.001)		0.014 (0.001)	-0.000 (0.001)
**Discrimination: C-index up to time 5**						
mean	0.546	0.578	0.547	0.555	0.552	0.556
bias (SE)		0.031 (0.000)	0.000 (0.000)		-0.003 (0.000)	0.001 (0.000)
**Discrimination: AUCt at time 5**						
mean	0.571	0.629	0.572	0.580	0.578	0.582
bias (SE)		0.058 (0.001)	0.000 (0.001)		-0.003 (0.001)	0.001 (0.001)
**Prediction error: scaled Brier score (%) at time 5**						
mean	-5.416	1.932	-5.421	-7.736	-7.149	-7.802
bias (SE)		7.347 (0.024)	-0.006 (0.080)		0.587 (0.082)	-0.066 (0.089)

AUCt: cumulative/dynamic area under the receiver operating characteristic curve.

eTable 6: Simulation results, Scenario 3, additive hazards model. Performance measures were obtained from the perfect validation data (true) and estimated from the observational validation data using the subset approach (subset) and using the proposed artificial censoring + inverse probability weighting estimators for assessing counterfactual performance (counterfactual). Results are averaged over 1000 simulation runs of validation datasets of size 3000.

	never treated			always treated		
	true	subset	counterfactual	true	subset	counterfactual
**Calibration: observed/expected ratio based on risk by time 5**						
mean	1.008	1.152	1.008	1.011	1.010	1.011
bias (SE)		0.145 (0.001)	-0.000 (0.001)		-0.000 (0.001)	0.000 (0.001)
**Discrimination: C-index up to time 5**						
mean	0.535	0.557	0.535	0.541	0.538	0.542
bias (SE)		0.023 (0.000)	0.000 (0.000)		-0.003 (0.000)	0.001 (0.000)
**Discrimination: AUCt at time 5**						
mean	0.554	0.596	0.554	0.560	0.557	0.561
bias (SE)		0.042 (0.001)	0.000 (0.001)		-0.003 (0.001)	0.001 (0.001)
**Prediction error: scaled Brier score (%) at time 5**						
mean	-0.001	-5.159	-0.030	-0.045	-0.125	-0.099
bias (SE)		-5.158 (0.088)	-0.029 (0.043)		-0.080 (0.048)	-0.054 (0.060)

AUCt: cumulative/dynamic area under the receiver operating characteristic curve.

eTable 7: Simulation results, Scenario 1, Cox model. Performance measures were obtained from the perfect validation data (true) and estimated from the observational validation data using the subset approach (subset) and using the proposed artificial censoring + inverse probability weighting estimators for assessing counterfactual performance (counterfactual). Results are averaged over 1000 simulation runs of validation datasets of size 3000.

	never treated			always treated		
	true	subset	counterfactual	true	subset	counterfactual
**Calibration: observed/expected ratio based on risk by time 5**						
mean	0.989	1.217	0.986	1.004	1.006	1.004
bias (SE)		0.227 (0.001)	-0.003 (0.002)		0.002 (0.002)	0.001 (0.003)
**Discrimination: C-index up to time 5**						
mean	0.600	0.626	0.600	0.608	0.608	0.609
bias (SE)		0.026 (0.000)	0.000 (0.000)		-0.001 (0.001)	0.001 (0.001)
**Discrimination: AUCt at time 5**						
mean	0.626	0.675	0.626	0.618	0.619	0.619
bias (SE)		0.050 (0.001)	0.001 (0.001)		0.001 (0.001)	0.001 (0.001)
**Prediction error: scaled Brier score (%) at time 5**						
mean	4.312	0.007	4.326	3.237	3.443	3.224
bias (SE)		-4.305 (0.110)	0.014 (0.081)		0.206 (0.041)	-0.013 (0.041)

AUCt: cumulative/dynamic area under the receiver operating characteristic curve.

eTable 8: Simulation results, Scenario 2, Cox model. Performance measures were obtained from the perfect validation data (true) and estimated from the observational validation data using the subset approach (subset) and using the proposed artificial censoring + inverse probability weighting estimators for assessing counterfactual performance (counterfactual). Results are averaged over 1000 simulation runs of validation datasets of size 3000.

	never treated			always treated		
	true	subset	counterfactual	true	subset	counterfactual
**Calibration: observed/expected ratio based on risk by time 5**						
mean	0.675	0.818	0.673	0.480	0.490	0.480
bias (SE)		0.143 (0.001)	-0.002 (0.001)		0.011 (0.001)	0.000 (0.001)
**Discrimination: C-index up to time 5**						
mean	0.600	0.626	0.600	0.608	0.608	0.609
bias (SE)		0.026 (0.000)	0.000 (0.000)		-0.001 (0.001)	0.001 (0.001)
**Discrimination: AUCt at time 5**						
mean	0.626	0.675	0.626	0.618	0.619	0.619
bias (SE)		0.050 (0.001)	0.001 (0.001)		0.001 (0.001)	0.001 (0.001)
**Prediction error: scaled Brier score (%) at time 5**						
mean	-29.033	-4.608	-29.228	-36.333	-38.210	-36.50
bias (SE)		24.425 (0.088)	-0.195 (0.190)		-1.877 (0.237)	-0.176 (0.230)

AUCt: cumulative/dynamic area under the receiver operating characteristic curve.

eTable 9: Simulation results, Scenario 3, Cox model. Performance measures were obtained from the perfect validation data (true) and estimated from the observational validation data using the subset approach (subset) and using the proposed artificial censoring + inverse probability weighting estimators for assessing counterfactual performance (counterfactual). Results are averaged over 1000 simulation runs of validation datasets of size 3000.

	never treated			always treated		
	true	subset	counterfactual	true	subset	counterfactual
**Calibration: observed/expected ratio based on risk by time 5**						
mean	0.988	1.214	0.986	0.965	0.971	0.967
bias (SE)		0.227 (0.001)	-0.002 (0.001)		0.006 (0.002)	0.001 (0.002)
**Discrimination: C-index up to time 5**						
mean	0.571	0.588	0.570	0.577	0.575	0.577
bias (SE)		0.018 (0.000)	-0.000 (0.000)		-0.001 (0.001)	0.001 (0.001)
**Discrimination: AUCt at time 5**						
mean	0.589	0.623	0.589	0.584	0.584	0.585
bias (SE)		0.034 (0.001)	-0.000 (0.001)		0.000 (0.001)	0.001 (0.001)
**Prediction error: scaled Brier score (%) at time 5**						
mean	-1.633	-6.951	-1.753	-0.340	-0.370	-0.334
bias (SE)		-5.317 (0.126)	-0.120 (0.113)		-0.030 (0.063)	0.006 (0.060)

AUCt: cumulative/dynamic area under the receiver operating characteristic curve.

eTable 10: Simulation results, Scenarios 4,5,6, additive hazards model. Bias (SE) of the performance measures obtained from the observational validation data using proposed artificial censoring + inverse probability weighting estimators for assessing counterfactual performance, compared to true performance obtained from the perfect validation data. Numbers displayed in bold indicate that results of the counterfactual performance measures had higher bias than those of the naive subset approach. Results are averaged over 1000 simulation runs of validation datasets of size 3000.

	never treated				always treated			
scenario	OE ratio	C-index	AUCt	Brier (%)	OE ratio	C-index	AUCt	Brier (%)
4a: Positivity violation: on average only 31 individuals untreated after time point 4								
	-0.007 (0.003)	-0.002 (0.001)	0.054 (0.004)	3.421 (0.383)	-0.001 (0.001)	0.000 (0.000)	0.001 (0.000)	0.014 (0.024)
4b: Positivity violation: on average only 73 individuals untreated after time point 4								
	-0.003 (0.002)	-0.001 (0.001)	0.021 (0.003)	1.164 (0.169)	-0.000 (0.001)	0.000 (0.000)	0.000 (0.000)	-0.010 (0.027)
5a: Exchangeability violation: weights do not use L								
	-0.032 (0.001)	-0.002 (0.000)	0.041 (0.001)	1.443 (0.037)	0.034 (0.001)	-0.003 (0.000)	-0.003 (0.001)	-0.250 (0.033)
5b: Exchangeability violation: weights use L_0 instead of L_k								
	-0.013 (0.001)	-0.001 (0.000)	-0.030 (0.001)	-1.225 (0.048)	0.000 (0.001)	0.001 (0.000)	0.001 (0.001)	-0.068 (0.042)
6a: Misspecified weights: weights use $\log(L_k)$ instead of L_k								
	-0.002 (0.001)	-0.001 (0.000)	0.001 (0.001)	-0.022 (0.028)	0.004 (0.001)	-0.001 (0.000)	-0.002 (0.001)	-0.195 (0.038)
6b: Misspecified weights: weights use L_k^2 instead of L_k								
	-0.012 (0.002)	0.006 (0.001)	0.058 (0.003)	4.186 (0.277)	0.006 (0.001)	-0.003 (0.000)	**-0.004 (0.001)**	**-0.305 (0.035)**
6c: Misspecified weights: L_k^2 in data generation, weights use L_k only								
	-0.014 (0.001)	-0.008 (0.000)	-0.015 (0.001)	0.032 (0.033)	**-0.023 (0.002)**	**0.013 (0.001)**	**0.016 (0.001)**	**1.373 (0.092)**
6d: Misspecified weights: weights use cauchit link in stead of logit link								
	0.001 (0.001)	0.002 (0.000)	0.003 (0.001)	0.299 (0.068)	0.000 (0.001)	0.001 (0.000)	0.001 (0.001)	-0.068 (0.042)

OE ratio: ratio of observed versus expected risk.

AUCt: cumulative/dynamic area under the receiver operating characteristic curve.

eTable 11: Simulation results, Scenarios 4,5,6, Cox model. Bias (SE) of the performance measures obtained from the observational validation data using proposed artificial censoring + inverse probability weighting estimators for assessing counterfactual performance, compared to true performance obtained from the perfect validation data. Numbers displayed in bold indicate that results of the counterfactual performance measures had higher bias than those of the naive subset approach. Results are averaged over 1000 simulation runs of validation datasets of size 3000.

	never treated				always treated			
scenario	OE ratio	C-index	AUCt	Brier (%)	OE ratio	C-index	AUCt	Brier (%)
4a: Positivity violation: on average only 25 individuals untreated after time point 4								
	-0.026 (0.006)	-0.000 (0.002)	0.034 (0.004)	3.603 (0.720)	0.000 (0.002)	0.000 (0.000)	0.000 (0.000)	0.015 (0.027)
4b: Positivity violation: on average only 73 individuals untreated after time point 4								
	-0.009 (0.003)	-0.001 (0.001)	0.007 (0.003)	0.742 (0.294)	0.001 (0.002)	0.000 (0.001)	0.000 (0.001)	0.015 (0.031)
5a: Exchangeability violation: weights do not use L								
	-0.149 (0.001)	-0.006 (0.000)	0.018 (0.001)	0.832 (0.064)	**0.114 (0.003)**	-0.001 (0.001)	0.001 (0.001)	0.206 (0.041)
5b: Exchangeability violation: weights use L_0 instead of L_k								
	-0.108 (0.001)	-0.003 (0.000)	-0.036 (0.001)	**-4.972 (0.109)**	0.001 (0.003)	0.001 (0.001)	0.001 (0.001)	-0.013 (0.041)
6a: Misspecified weights: weights use $\log(L_k)$ instead of L_k								
	-0.009 (0.001	-0.001 (0.000)	-0.001 (0.001)	-0.096 (0.078)	-0.001 (0.003)	**0.002 (0.001)**	**0.002 (0.001)**	0.038 (0.041)
6b: Misspecified weights: weights use L_k^2 instead of L_k								
	-0.152 (0.001)	-0.004 (0.000)	0.024 (0.001)	1.212 (0.065)	**0.105 (0.003)**	**-0.004 (0.001)**	**-0.002 (0.001)**	-0.030 (0.038)
6c: Misspecified weights: L_k^2 in data generation, weights use L_k only								
	-0.088 (0.002)	-0.022 (0.000)	**-0.027 (0.001)**	-0.405 (0.087)	**-0.023 (0.002)**	**0.018 (0.001)**	**0.020 (0.001)**	**1.045 (0.046)**
6d: Misspecified weights: weights use cauchit link instead of logit link								
	0.008 (0.002	0.004 (0.000)	0.006 (0.001)	0.636 (0.096)	0.001 (0.003)	0.001 (0.001)	0.001 (0.001)	-0.013 (0.041)

OE ratio: ratio of observed versus expected risk.

AUCt: cumulative/dynamic area under the receiver operating characteristic curve.

eTable 12: Liver transplant application, development data D^{dev}: Summary of covariates as recorded at time zero for the $n = 116797$ person-landmark observations in the development data, where time zero is the time of transplant for person-landmark observations in D_1^{dev} and the landmark time for person-landmark observations in D_0^{dev}. Summaries of numbers in the 11 regions are omitted. The numbers in each region ranged from 3660 (3.1%) (region 6) to 18460 (15.8%) (region 5).

(a) Time-fixed variables

		n (%) or Mean (SD)
Sex	Male	78038 (66.8)
	Female	38759 (33.2)
Ethnicity	Asian	4227 (3.6)
	Black	9303 (8.0)
	Other	1999 (1.7)
	White	101268 (86.7)
Blood group	A	44842 (38.4)
	AB	3666 (3.1)
	B	13848 (11.9)
	O	54441 (46.6)
Disease group	Alcoholic Cirrhosis	40360 (34.6)
	HBV Cirrhosis HBV	2162 (1.9)
	HCV Cirrhosis HCV	26975 (23.1)
	Cryptogenic	383 (0.3)
	HCC	15022 (12.9)
	NASH	23357 (20.0)
	PBC	3428 (2.9)
	PSC	5110 (4.4)
Diabetes	No	79116 (67.7)
	Yes	37681 (32.3)
BMI	mean (SD)	29.33 (5.86)

(b) Time-dependent variables (as recorded at time zero)

		n (%) or Mean (SD)
Age	mean (SD)	58.90 (8.99)
CKD	No	78734 (67.4)
	Yes	38063 (32.6)
Dialysis	No	108797 (93.2)
	Yes	8000 (6.8)
MELD-NA score	mean (SD)	15.59 (9.22)
Albumin	mean (SD)	3.24 (0.66)
Sodium	mean (SD)	136.81 (4.44)
Creatinine	mean (SD)	1.32 (1.26)
Bilirubin	mean (SD)	3.92 (6.29)
INR	mean (SD)	1.50 (0.64)
Ascites	Absent	37716 (32.3)
	Slight	55118 (47.2)
	Moderate	23963 (20.5)
Encephalopathy	1-2	59185 (50.7)
	3-4	6097 (5.2)
	None	51515 (44.1)
Child Pugh Score grade	A	26263 (22.5)
	B	51439 (44.0)
	C	39095 (33.5)
Number of tumours	0	89570 (76.7)
	1	23942 (20.5)
	≥ 2	3285 (2.8)
Exception points	No	87172 (74.6)
	Yes	29625 (25.4)
Exception points HCC	No	105841 (90.6)
	Yes	10956 (9.4)

eTable 13: Liver transplant application, validation data D^{val}: Summary of covariates as recorded at time zero for the $n = 49966$ person-landmark observations in the validation data, where time zero is the time of transplant for person-landmark observations in D_1^{val} and the landmark time for person-landmark observations in D_0^{val}. Summaries of numbers in the 11 regions are omitted. The numbers in each region ranged from 1529 (3.1%) (region 6) to 7688 (15.4%) (region 5).

(a) Time-fixed variables

		n (%) or Mean (SD)
Sex	Male	33529 (67.1)
	Female	16437 (32.9)
Ethnicity	Asian	1767 (3.5)
	Black	3894 (7.8)
	Other	853 (1.7)
	White	43452 (87.0)
Blood group	A	19141 (38.3)
	AB	1592 (3.2)
	B	5847 (11.7)
	O	23386 (46.8)
Disease group	Alcoholic Cirrhosis	17421 (34.9)
	HBV Cirrhosis	931 (1.9)
	HCV Cirrhosis	11539 (23.1)
	Cryptogenic	160 (0.3)
	HCC	6225 (12.5)
	NASH	10063 (20.1)
	PBC	1426 (2.9)
	PSC	2201 (4.4)
Diabetes	No	33958 (68.0)
	Yes	16008 (32.0)
BMI	mean (SD)	29.32 (5.82)

(b) Time-dependent variables (as recorded at time zero)

		n (%) or Mean (SD)
Age	mean (SD)	58.67 (9.11)
CKD	No	33603 (67.3)
	Yes	16363 (32.7)
Dialysis	No	46486 (93.0)
	Yes	3480 (7.0)
MELD-NA score	mean (SD)	15.62 (9.19)
Albumin	mean (SD)	3.25 (0.66)
Sodium	mean (SD)	136.79 (4.45)
Creatinine	mean (SD)	1.33 (1.28)
Bilirubin	mean (SD)	3.88 (6.20)
INR	mean (SD)	1.50 (0.65)
Ascites	Absent	16079 (32.2)
	Slight	23485 (47.0)
	Moderate	10402 (20.8)
Encephalopathy	1-2	25377 (50.8)
	3-4	2627 (5.3)
	None	21962 (44.0)
Child Pugh Score grade	A	11176 (22.4)
	B	22161 (44.4)
	C	16629 (33.3)
Number of tumours	0	38351 (76.8)
	1	10200 (20.4)
	$\geq$2	1415 (2.8)
Exception points	0	37283 (74.6)
	1	12683 (25.4)
Exception points HCC	0	45221 (90.5)
	1	4745 (9.5)

eTable 14: Liver transplant application: Summary of numbers of events and censorings in the validation datasets created to mimic the "no transplant" and "transplant" strategies.

	No transplant: V^0	Transplant: V^1
Number of person-landmark observations	49966	49966
Number of unique individuals	27929	27929
Number of person-landmark observations artificially censored due to deviation from strategy	24439	42820
Number of events (composite outcome)	7221	777
Number censored due to removal from waitlist due to improvement or for "other" reasons	7883	-
Number administratively censored	10423	6369
Total person-landmark-observation-years of follow-up	35478	13126

eTable 15: Liver transplant application: Evaluation of model performance using the subset approach.

	Strategy	
	No transplant	Transplant
Calibration: observed/expected ratio based on risk by 3 years	1.058	1.010
Discrimination: C-index up to 3 years	0.756	0.587
Discrimination: AUCt at 3 years	0.783	0.578
Prediction error: scaled Brier score (%) at 3 years	18.2	0.46

AUCt: cumulative/dynamic area under the receiver operating characteristic curve.

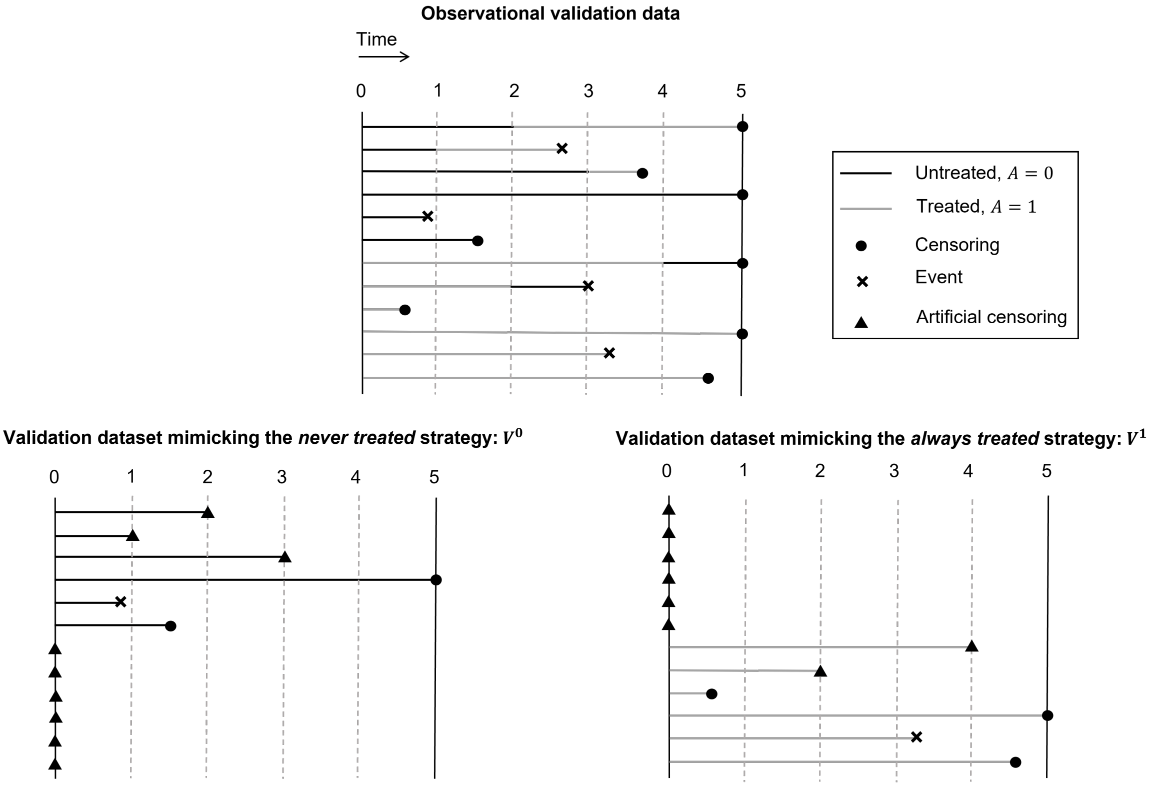

eFigure 1: Illustration of how the validation datasets $V^{\underline{a}_0=0}$ and $V^{\underline{a}_0=1}$ are created from the observational validation data. The original data depicts observed follow-up for 12 individuals, with 5 visit times and administrative censoring at time 5. Treatment status A_t is observed at times $t = 0, 1, 2, 3, 4$ and assumed to be constant between visits. For the *always treated* strategy, individuals are artificially censored at the time t at which they have $A_t = 0$, if this occurs before their end of follow-up. Individuals with $A_0 = 0$ are censored immediately at time 0, and those with $A_0 = 1$ who later switch to $A_t = 0$ are censored at the time of the switch. For the *never treated* strategy, individuals in the validation data are artificially censored when they deviate from the *never treated* strategy, with some individuals (those with $A_0 = 1$) being censored at time 0.

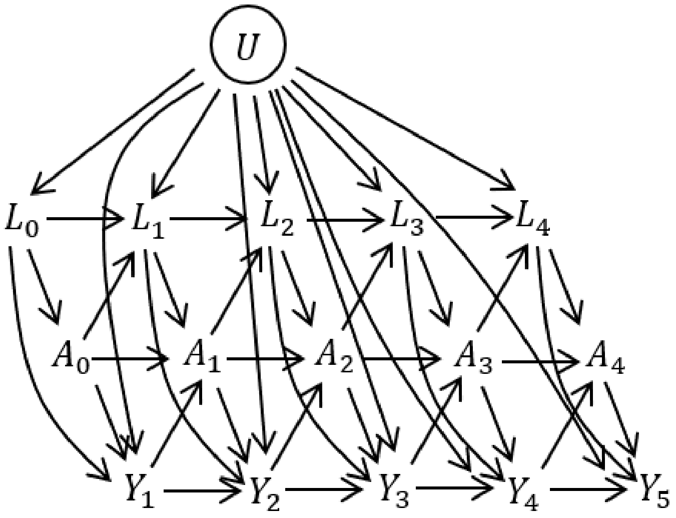

eFigure 2: Directed acyclic graph (DAG) illustrating the data generating mechanism for the simulation study, for treatment A, time-dependent covariates L, and discrete time outcome Y. The DAG is illustrated for a discrete-time setting where $Y_k = I(k-1 \leq T < k)$ is an indicator of whether the event occurs between visits $k-1$ and k. If the DAG is extended by adding a series of small time intervals between each visit, at which events are observed, then we approach the continuous time setting. The covariates L_k are time-dependent confounders as they inform treatment initiation or continuation, are predictive of the outcome, and are also affected by past treatment.

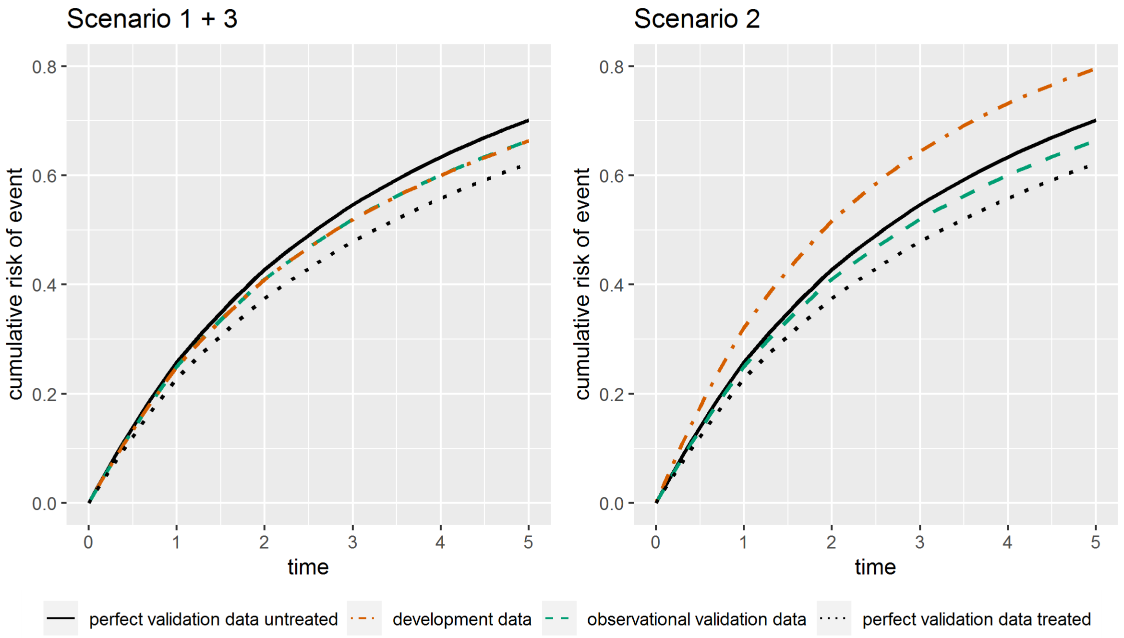

eFigure 3: Marginal risk distribution estimated from the development datasets, observational validation datasets and perfect counterfactual *never treated* and *always treated* datasets generated using the additive hazards model, main Scenarios 1-3. The curves are constructed by averaging over the (one minus) survival curves estimated by the Kaplan-Meier estimator in each of the 1000 simulation runs.

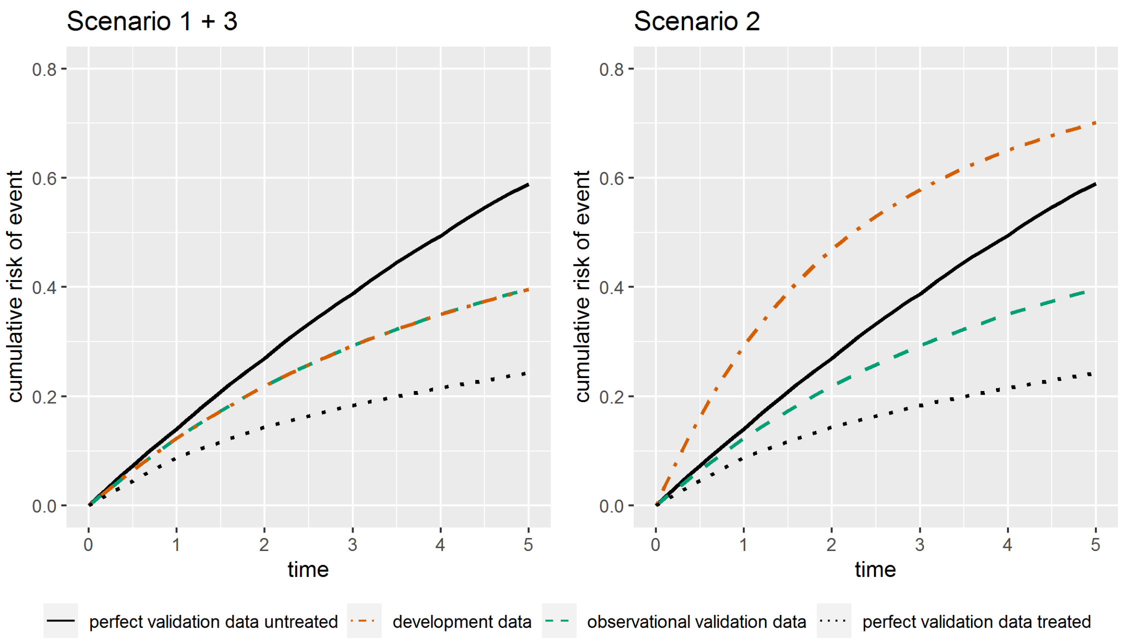

eFigure 4: Marginal risk distribution estimated from the development datasets, observational validation datasets and perfect counterfactual *never treated* and *always treated* datasets generated using the Cox model, main Scenarios 1-3. The curves are constructed by averaging over the (one minus) survival curves estimated by the Kaplan-Meier estimator in each of the 1000 simulation runs.

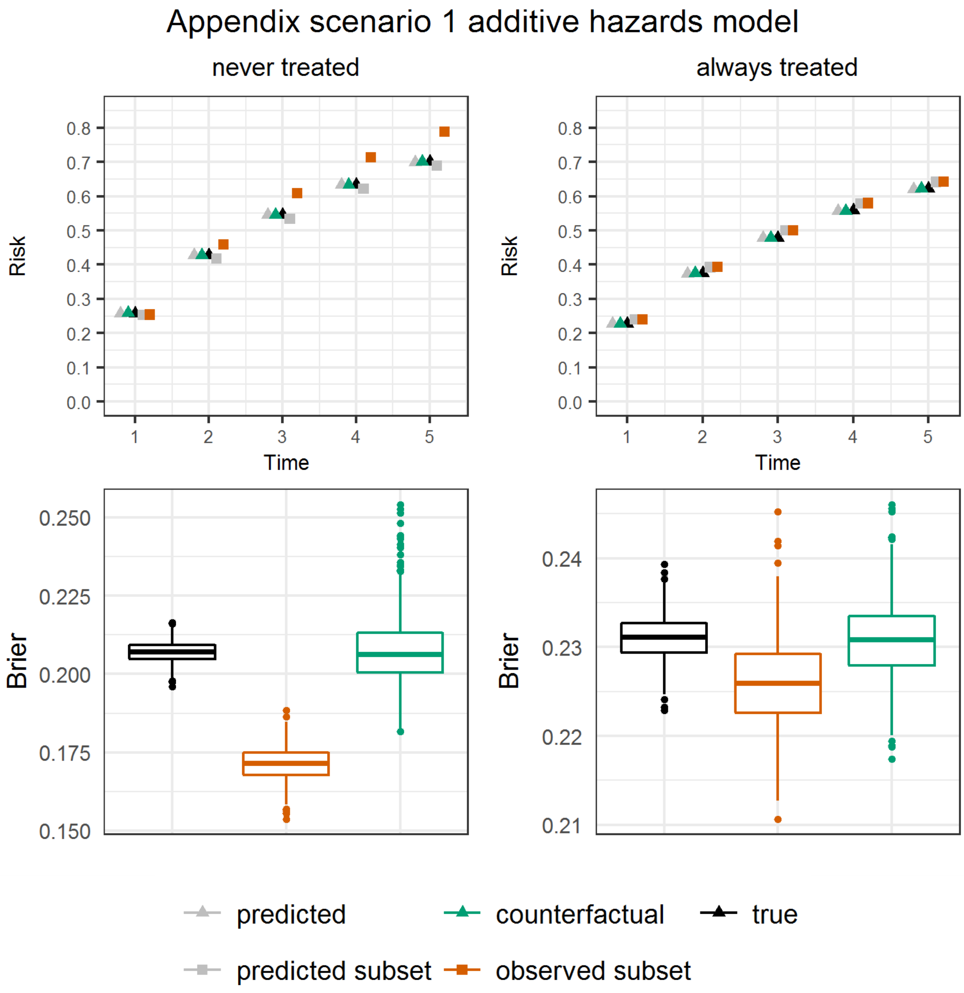

eFigure 5: Simulation results: additive hazards model Scenario 1. Left panel: for the *never treated* strategy. Right panel: for the *always treated* strategy. Top row: outcome proportions over time estimated by the prediction model (grey triangles), observed in the perfect validaiton data (black triangles) and estimated from the observational validation data using the proposed artificial censoring + inverse probability weighting estimators for counterfactual performance assessment (green triangles). Estimated and observed outcome proportions using the subset approach are depicted with grey and orange squares. Bottom row: unscaled Brier score at time 5.

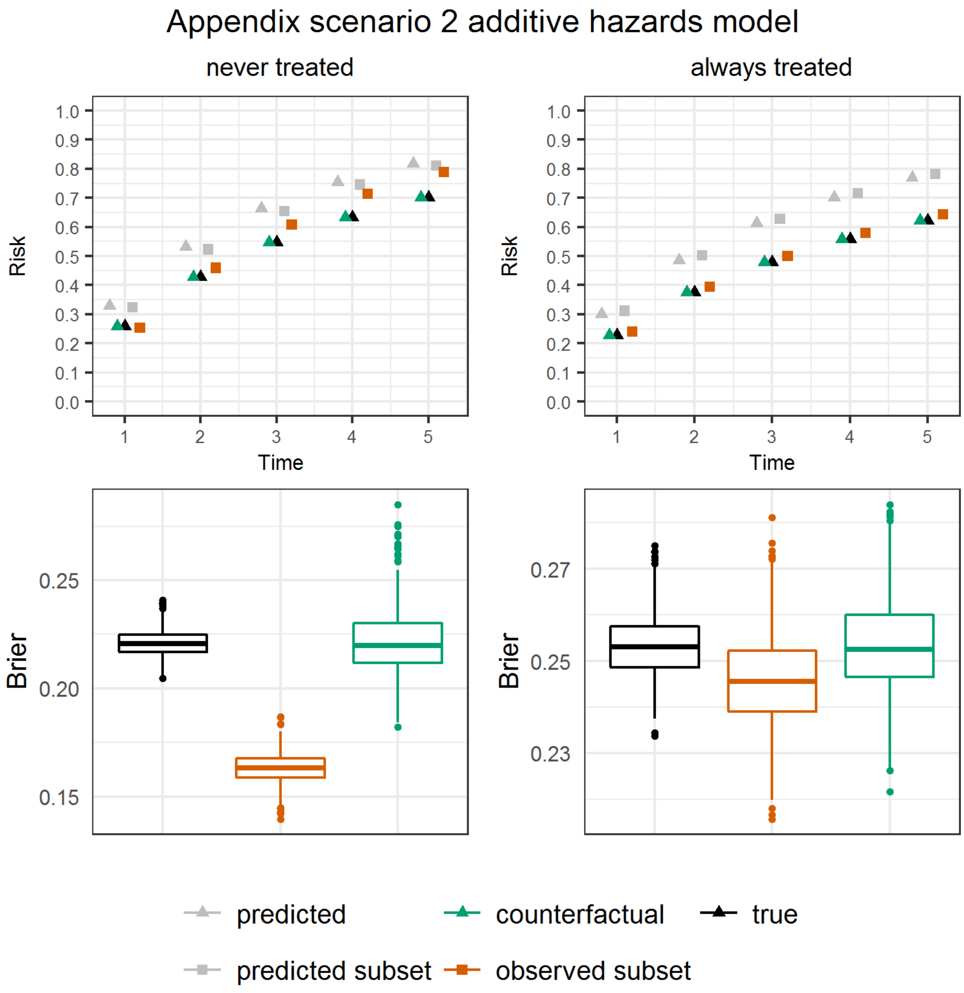

eFigure 6: Simulation results: additive hazards model Scenario 2. Left panel: for the *never treated* strategy. Right panel: for the *always treated* strategy. Top row: outcome proportions over time estimated by the prediction model (grey triangles), observed in the perfect validaiton data (black triangles) and estimated from the observational validation data using the proposed artificial censoring + inverse probability weighting estimators for counterfactual performance assessment (green triangles). Estimated and observed outcome proportions using the subset approach are depicted with grey and orange squares. Bottom row: unscaled Brier score at time 5.

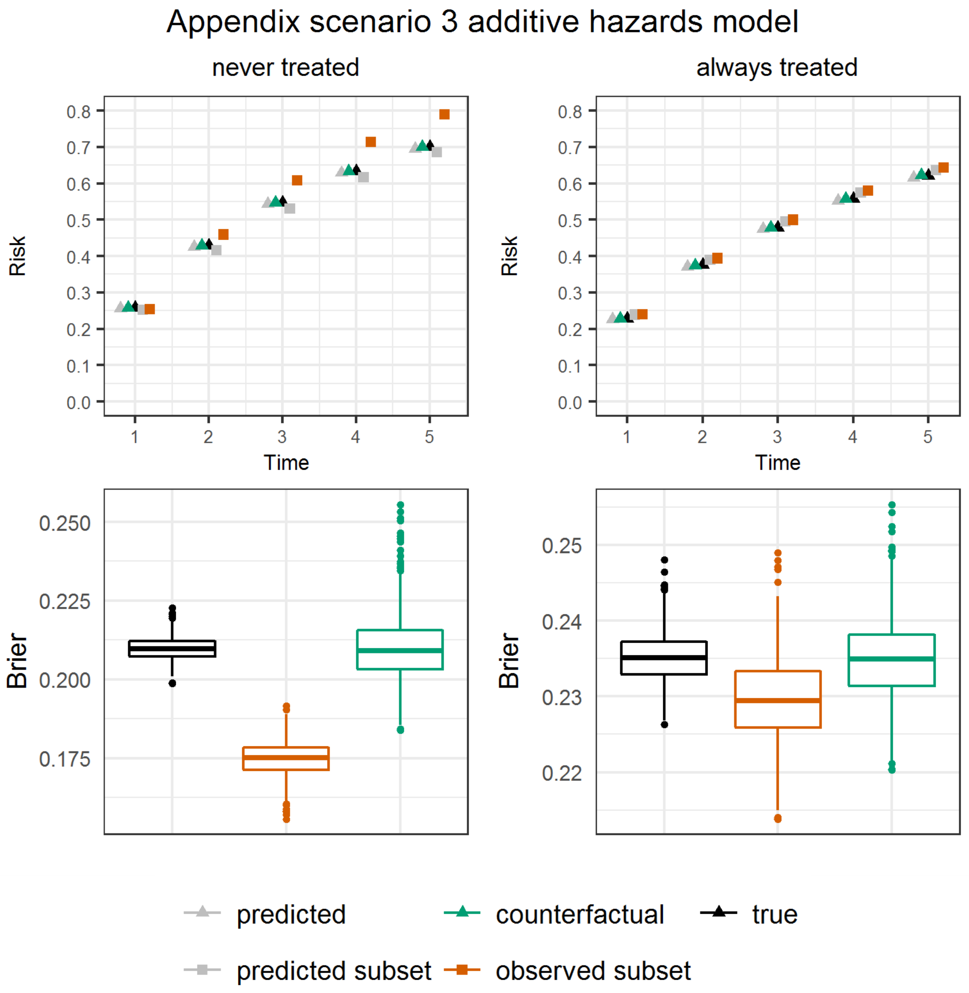

eFigure 7: Simulation results: additive hazards model Scenario 3. Left panel: for the *never treated* strategy. Right panel: for the *always treated* strategy. Top row: outcome proportions over time estimated by the prediction model (grey triangles), observed in the perfect validaiton data (black triangles) and estimated from the observational validation data using the proposed artificial censoring + inverse probability weighting estimators for counterfactual performance assessment (green triangles). Estimated and observed outcome proportions using the subset approach are depicted with grey and orange squares. Bottom row: unscaled Brier score at time 5.

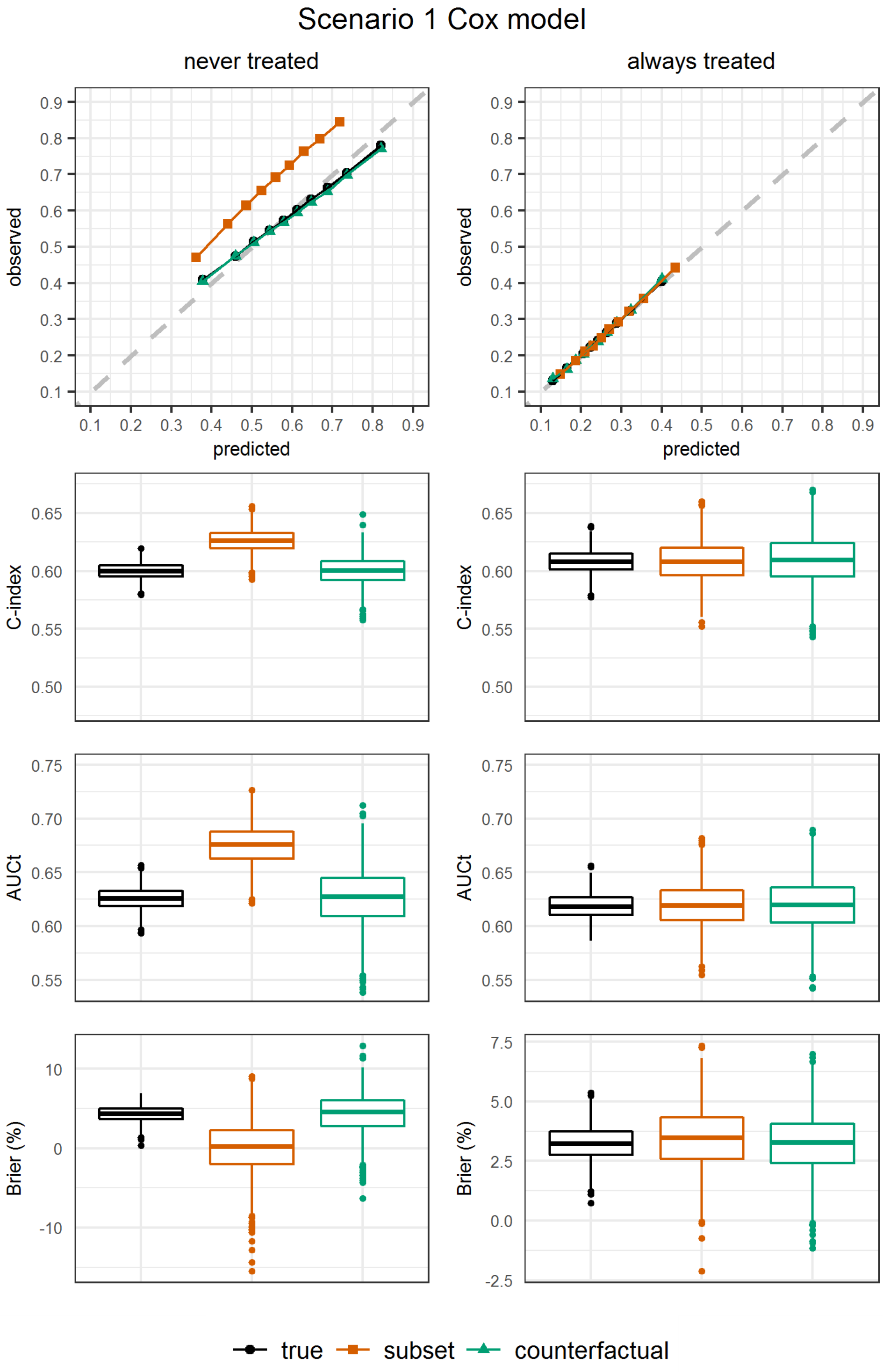

eFigure 8: Simulation results: Cox model Scenario 1. Left panel: for the *never treated* strategy. Right panel: for the *always treated* strategy. Performance measures were obtained from the perfect validation data (black dots) and estimated from the observational validation data using the subset approach (orange squares) and using the proposed artificial censoring + inverse probability weighted estimators of counterfactual performance (green triangles). Top row: calibration plot showing observed outcome proportions against mean estimated risks by time 5 within tenths of the estimated risks. Second row: c-index truncated at time 5. Third row: cumulative/dynamic area under the receiver operating characteristic curve at time 5. Bottom row: scaled Brier score at time 5.

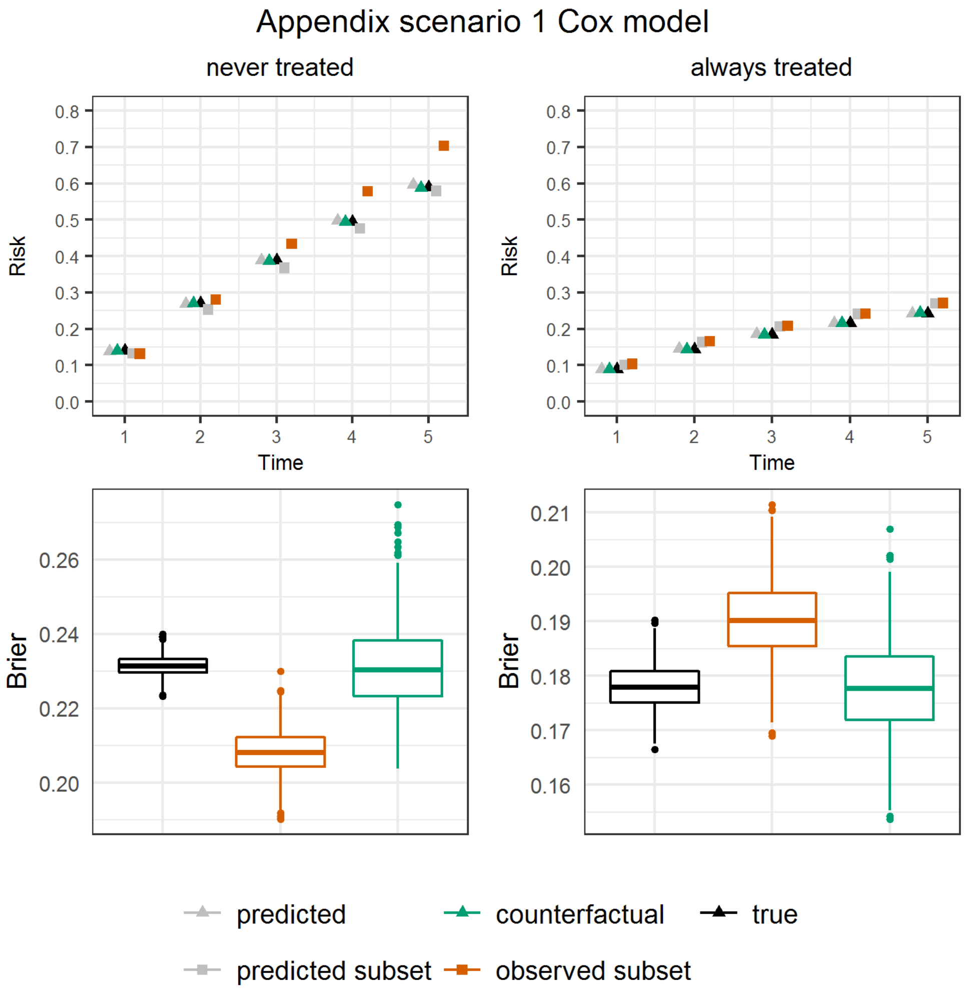

eFigure 9: Simulation results: Cox model Scenario 1. Left panel: for the *never treated* strategy. Right panel: for the *always treated* strategy. Top row: outcome proportions over time estimated by the prediction model (grey triangles), observed in the perfect validaiton data (black triangles) and estimated from the observational validation data using the proposed artificial censoring + inverse probability weighting estimators for counterfactual performance assessment (green triangles). Estimated and observed outcome proportions using the subset approach are depicted with grey and orange squares. Bottom row: unscaled Brier score at time 5.

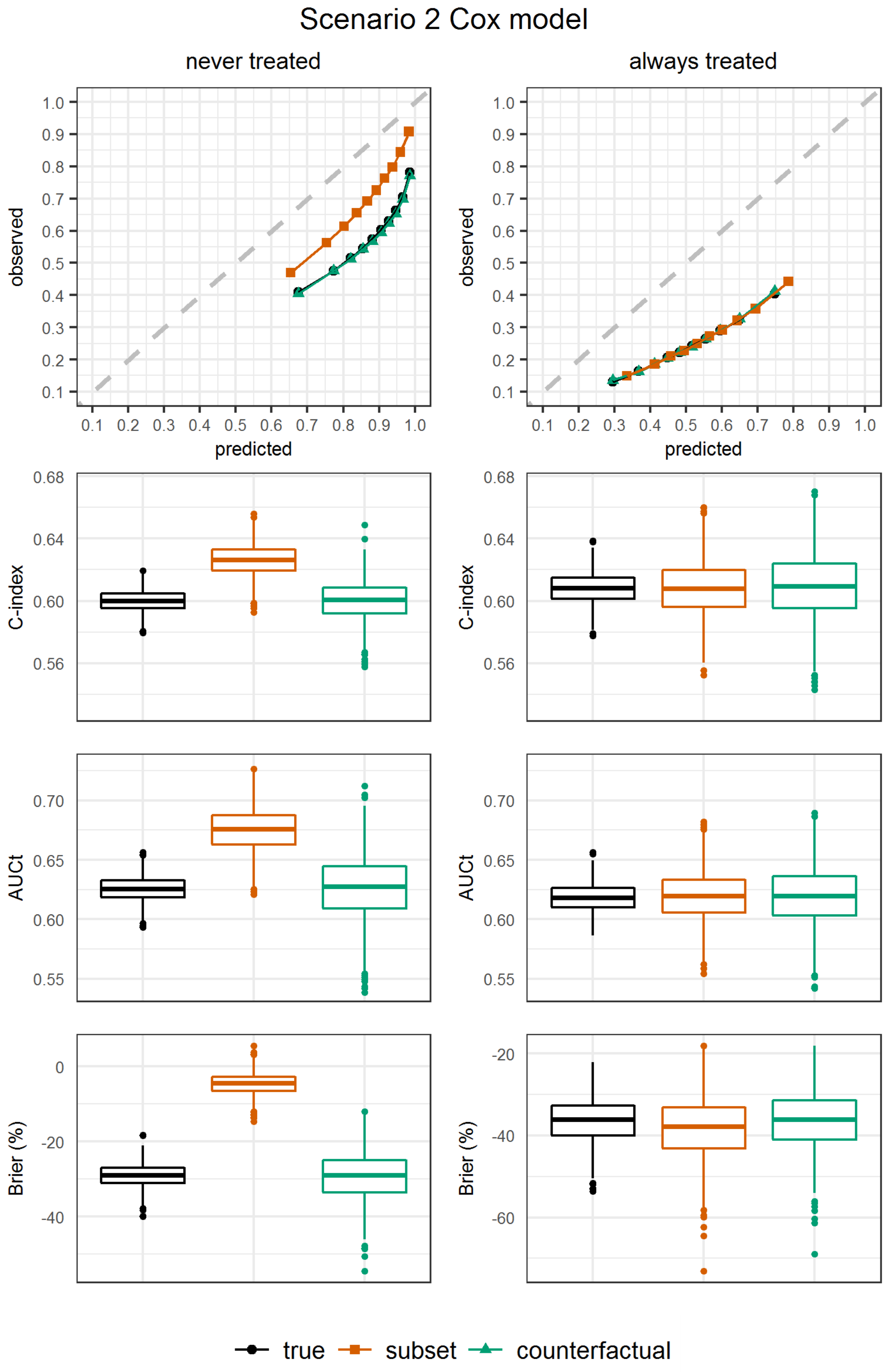

eFigure 10: Simulation results: Cox model Scenario 2. Left panel: for the *never treated* strategy. Right panel: for the *always treated* strategy. Performance measures were obtained from the perfect validation data (black dots) and estimated from the observational validation data using the subset approach (orange squares) and using the proposed artificial censoring + inverse probability weighted estimators of counterfactual performance (green triangles). Top row: calibration plot showing observed outcome proportions against mean estimated risks by time 5 within tenths of the estimated risks. Second row: c-index truncated at time 5. Third row: cumulative/dynamic area under the receiver operating characteristic curve at time 5. Bottom row: scaled Brier score at time 5.

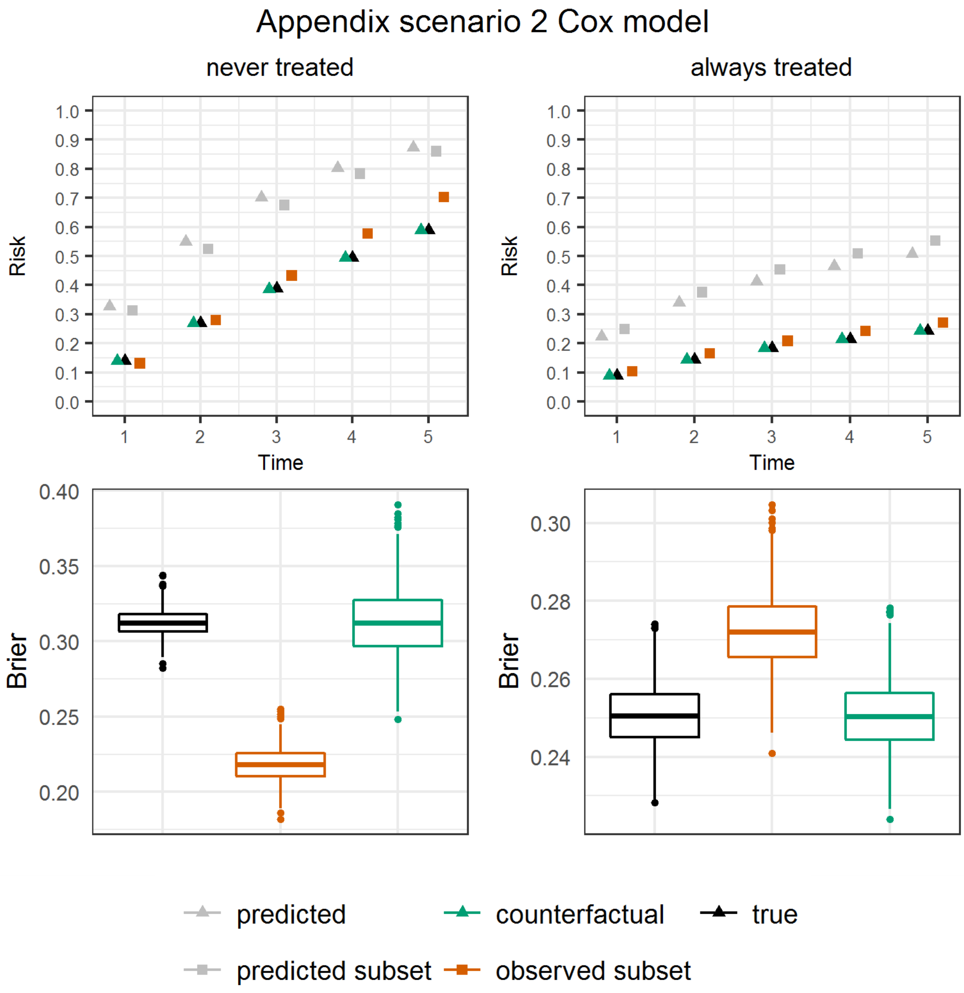

eFigure 11: Simulation results: Cox model Scenario 2. Left panel: for the *never treated* strategy. Right panel: for the *always treated* strategy. Top row: outcome proportions over time estimated by the prediction model (grey triangles), observed in the perfect validaiton data (black triangles) and estimated from the observational validation data using the proposed artificial censoring + inverse probability weighting estimators for counterfactual performance assessment (green triangles). Estimated and observed outcome proportions using the subset approach are depicted with grey and orange squares. Bottom row: unscaled Brier score at time 5.

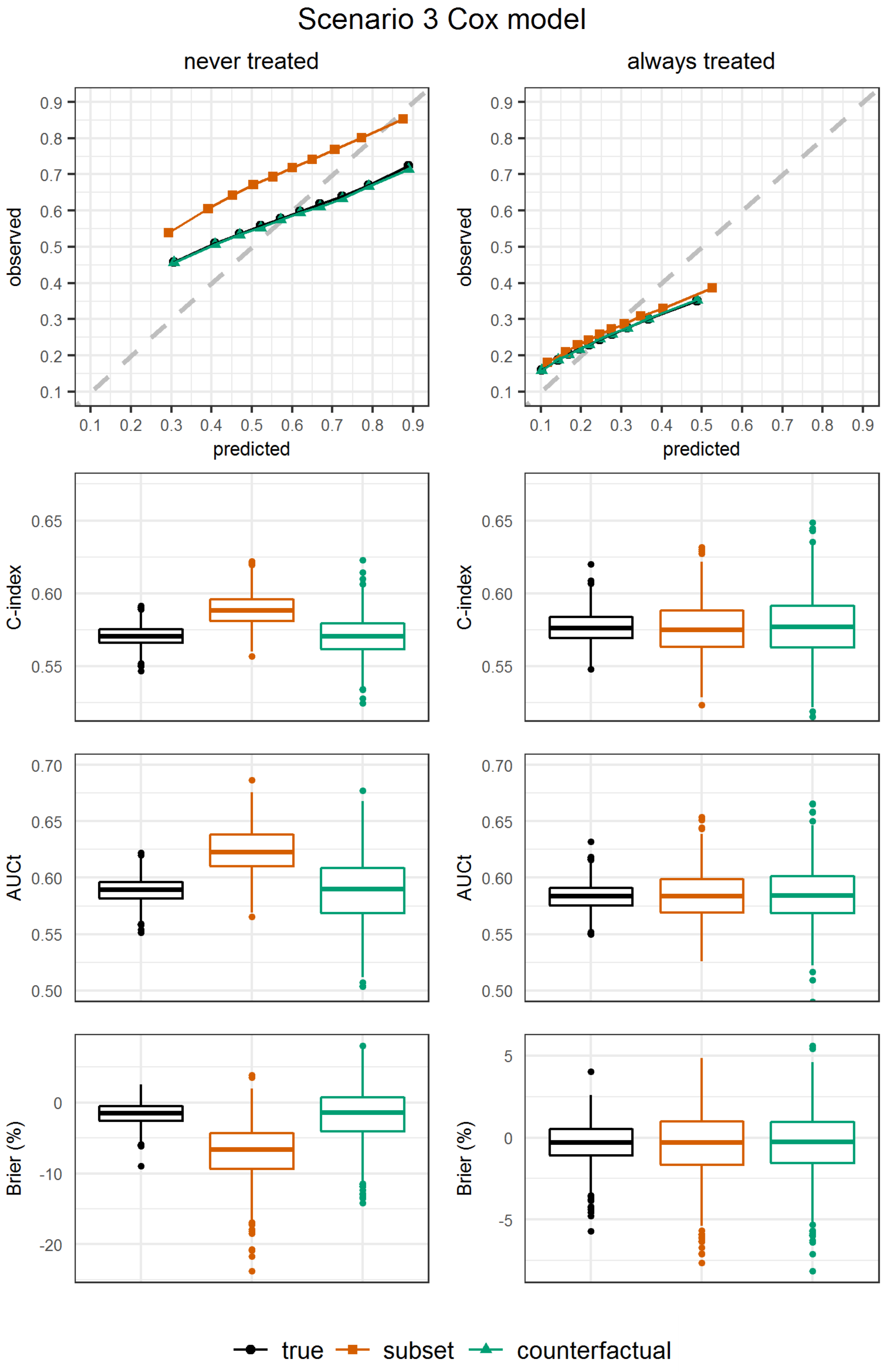

eFigure 12: Simulation results: Cox model Scenario 3. Left panel: for the *never treated* strategy. Right panel: for the *always treated* strategy. Performance measures were obtained from the perfect validation data (black dots) and estimated from the observational validation data using the subset approach (orange squares) and using the proposed artificial censoring + inverse probability weighted estimators of counterfactual performance (green triangles). Top row: calibration plot showing observed outcome proportions against mean estimated risks by time 5 within tenths of the estimated risks. Second row: c-index truncated at time 5. Third row: cumulative/dynamic area under the receiver operating characteristic curve at time 5. Bottom row: scaled Brier score at time 5.

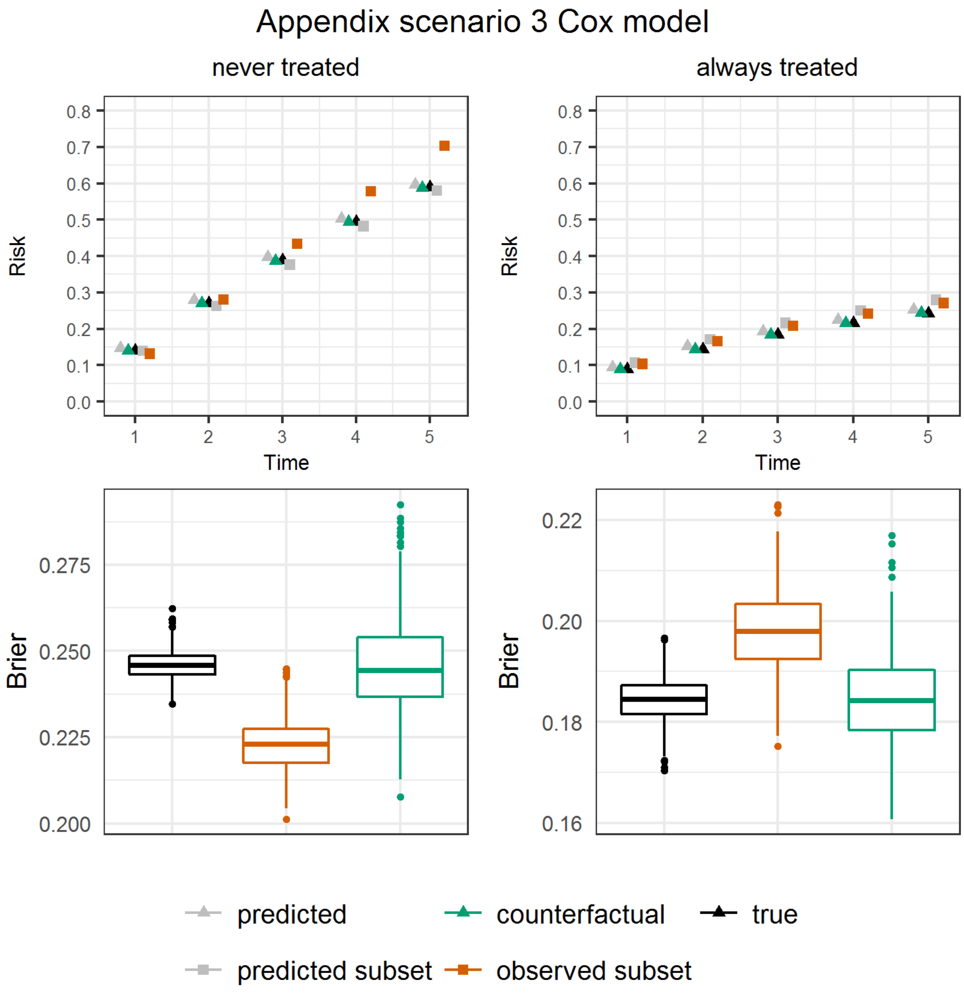

eFigure 13: Simulation results: Cox model Scenario 3. Left panel: for the *never treated* strategy. Right panel: for the *always treated* strategy. Top row: outcome proportions over time estimated by the prediction model (grey triangles), observed in the perfect validaiton data (black triangles) and estimated from the observational validation data using the proposed artificial censoring + inverse probability weighting estimators for counterfactual performance assessment (green triangles). Estimated and observed outcome proportions using the subset approach are depicted with grey and orange squares. Bottom row: unscaled Brier score at time 5.

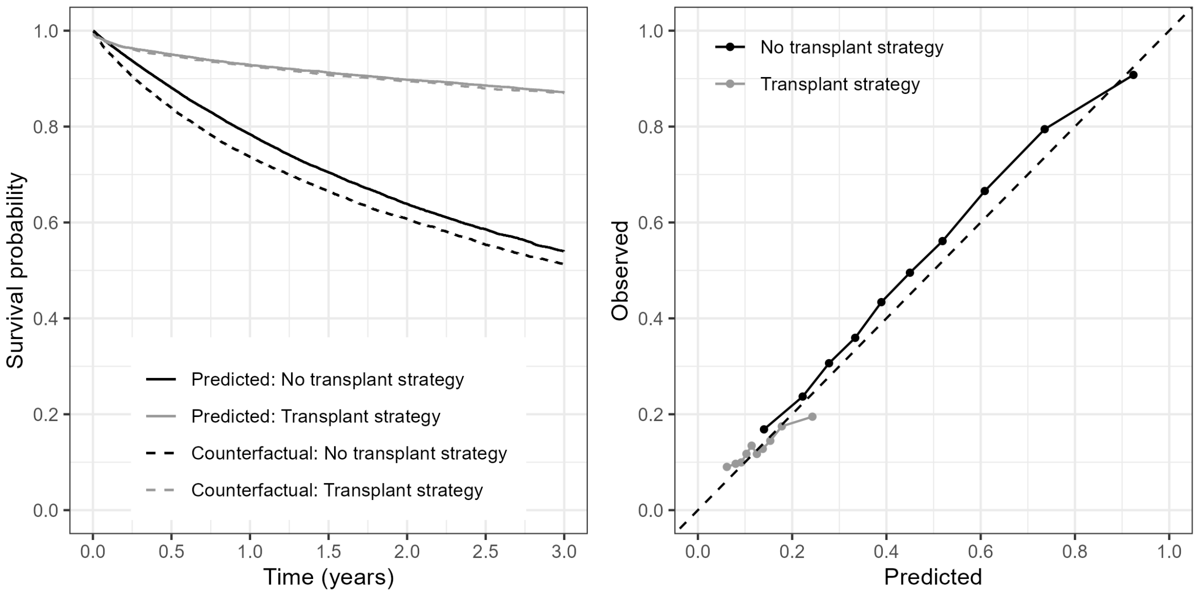

eFigure 14: Liver transplant application: Calibration of estimated risks under the *no transplant* and *transplant* strategies. Left: Plot showing the mean estimated survival curves up to three years under the two transplant strategies (solid lines), and the corresponding observed survival curves (dashed lines), obtained using the subset approach. Right: Plot of mean observed outcome proportions by 3 years (obtained using the subset approach) against mean estimated risk by 3 years within 10 equal-sized groups of estimated risk under the two transplant strategies, showing the line of equality (dashed line).
